# Identification of biomarker microRNAs for predicting the response of colorectal cancer to neoadjuvant chemoradiotherapy based on microRNA regulatory network

**DOI:** 10.18632/oncotarget.13659

**Published:** 2016-11-26

**Authors:** Yaqun Zhu, Qiliang Peng, Yuxin Lin, Li Zou, Peipei Shen, Feifei Chen, Ming Min, Li Shen, Jiajia Chen, Bairong Shen

**Affiliations:** ^1^ Department of Radiotherapy and Oncology, Second Affiliated Hospital of Soochow University, Suzhou, China; ^2^ Institute of Radiotherapy and Oncology, Soochow University, Suzhou, China; ^3^ Suzhou Key Laboratory for Radiation Oncology, Suzhou, China; ^4^ Center for Systems Biology, Soochow University, Suzhou, China; ^5^ Institute of Biological Sciences and Biotechnology, Donghua University, Shanghai, China; ^6^ School of Chemistry, Biology and Material Engineering, Suzhou University of Science and Technology, Suzhou, China; ^7^ Key laboratory of Systems Biology, Shanghai Institute of Biological Sciences, Chinese Academy of Sciences, Shanghai, China

**Keywords:** biomarker microRNA, colorectal cancer, neoadjuvant chemoradiotherapy, microRNA regulatory network, bioinformatics model

## Abstract

Preoperative radiotherapy or chemoradiotherapy has become a standard procedure for treatment of patients with locally advanced colorectal cancer (CRC). However, patients’ responses to treatment are different and personalized. MicroRNAs (miRNAs) are promising biomarkers for predicting personalized responses. In this study, we collected 30 publicly reported miRNAs associated with chemoradiotherapy of CRC. We extracted 46 differentially expressed miRNAs from samples of responders and non-responders to preoperative radiotherapy from the Gene Expression Omnibus dataset (Student's *t* test, *p-value* < 0.05 and |fold-change| > 2). We performed a systematic and integrative bioinformatics analysis to identify biomarker miRNAs for prediction of CRC responses to chemoradiotherapy. Using the bioinformatics model, miR-198, miR-765, miR-671-5p, miR-630, miR-371-5p, miR-575, miR-202, miR-483-5p and miR-513a-5p were screened as putative biomarkers for treatment response. Literature validation and functional enrichment analysis were exploited to confirm the reliability of the predicted miRNAs. Quantitative polymerase chain reaction showed that seven of the candidates were significantly differentially expressed between radiosensitive and insensitive CRC cell lines. The unique target genes of miR-198 and miR-765 were altered significantly upon transfection of specific miRNA mimics in the radiosensitive cell line. These results demonstrated the predictive power of our model and suggested that miR-198, miR-765, miR-630, miR-371-5p, miR-575, miR-202 and miR-513a-5p could be used for predicting the response of CRC to preoperative chemoradiotherapy.

## INTRODUCTION

Colorectal cancer (CRC) is one of the most common cancers worldwide and is the second most common in women and third most common in men [1]. CRC is easily confused with hemorrhoids and polyps and is often diagnosed at an advanced stage [2]. As a result, the mortality of CRC has been increasing and accurate diagnosis and treatment of CRC are urgently needed [1]. In recent years, preoperative radiotherapy or chemoradiotherapy has become a standard procedure for the treatment of patients with locally advanced rectal cancer. However, patients’ responses to preoperative chemoradiotherapy are personalized and not all patients benefit from this treatment [3].

Many genetic and molecular biomarkers have the potential to predict the responses of CRC to preoperative chemoradiotherapy, including p53, epidermal growth factor receptor, thymidylate synthase, p21, Bcl-2, and Bax [4]. Given the current controversial results, still no specific biomarkers can effectively distinguish responders from non-responders to preoperative radiotherapy or chemoradiotherapy [5]. New approaches for predicting the CRC response to preoperative chemoradiotherapy are urgently needed. MicroRNAs (miRNAs) are small noncoding RNAs that play important roles in regulating gene expression [6], cell growth, differentiation and apoptosis, as well as cancer development and metastasis. Abnormal expression of miRNAs is related to human carcinogenesis [7, 8]. Therefore, miRNAs are good candidates as diagnostic, therapeutic or prognostic biomarkers for cancer [9].

Thus far, a lot of research has been conducted on the response of cancer to radiation, including prostate, cervical, breast and colorectal cancer. It is encouraging that several putative miRNA biomarkers are reportedly associated with cancer response to preoperative chemoradiotherapy [10]. At present, it is still hard to explain the difference in radiosensitivity difference among patients. Routine methods used in biomarker discovery are based on exploring the outlier miRNAs and then performing reverse transcription polymerase chain reaction (RT-PCR) and bioinformatics analysis for validation. Different experimental platforms vary greatly and the topological features in miRNA–mRNA networks have not yet been investigated. Meanwhile, no studies have described biomarker miRNAs and characterized their functions and network features.

**Figure 1:**
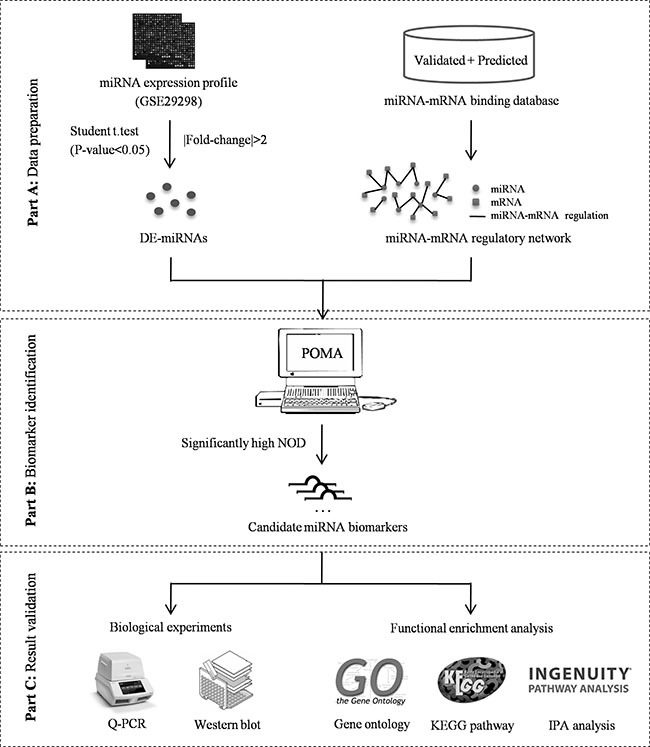
Schematic pipeline of this study

Considering the heterogeneity and complexity of carcinogenesis, miRNA biomarker discovery should be based on a systems biological analysis that combines miRNA and mRNA expression profiles and the interactions between miRNAs and mRNAs. In this study, we applied an integrated bioinformatics model termed POMA (Pipeline of Outlier MicroRNA Analysis) to investigate the toplogical feature of known biomarker miRNAs in human miRNA–mRNA network, and to predict novel biomarker miRNAs that could differentiate CRC responders from non-responders to neoadjuvant chemoradiotherapy. The model was enriched for frail sites in miRNA–mRNA networks and focused on the independent regulatory power of miRNAs. The parameter NOD (novel out degree), which was equivalent to the number of genes that were uniquely regulated by an individual miRNA, was defined to quantify the power. According to our previous studies, miRNAs with high NOD values are likely to be biomarkers, and applications in biomarker discovery for prostate cancer [11, 12] and pediatric acute myeloid leukemia [13] have confirmed their predictive ability. Here, we extended application of the model to the biomarker miRNA discovery for prediction of CRC responses to chemoradiotherapy, and the identified miRNAs were validated experimentally. The schematic pipeline of this study is presented in Figure 1.

## RESULTS

### Topological and functional role of known chemoradiotherapy associated miRNAs

Thirty miRNAs (Table 1) associated with CRC responses to preoperative chemoradiotherapy were mined and collected from PubMed by keywords searching.

**Table 1:** Details of CRC chemoradiotherapy associated miRNAs collected from literature

Reported ID	Official ID	Year	Expression level	Sample type	Experimental method	Reference	NOD value
miR-630	miR-630	2011	up	Tissue	qRT-PCR	[14]	5
miR-622	miR-622	2011	up	Tissue	qRT-PCR	[14]	1
miR-483-5p	miR-483-5p	2011	up	Tissue	qRT-PCR	[14]	4
miR-125a-3p	miR-125a-3p	2011	up	Tissue	qRT-PCR	[14]	2
miR-188-5p	miR-188-5p	2011	up	tissue	qRT-PCR	[14]	1
miR-671-5p	miR-671-5p	2011	up	tissue	qRT-PCR	[14]	10
miR-765	miR-765	2011	up	tissue	qRT-PCR	[14]	10
miR-196b	miR-196b-5p	2012	up	tissue	TLDA	[57]	12
let-7e	let-7e-5p	2012	up	tissue	TLDA	[57]	2
miR-223	miR-223-3p	2012	up	tissue	qRT-PCR	[58]	9
let-7a	let-7a-5p	2013	up	cell lines	qRT-PCR	[59]	5
miR-21	miR-21-5p	2013	up	cell lines	qRT-PCR	[60]	38
miR-221	miR-221-3p	2013	up	cell lines	qRT-PCR	[61]	11
miR-132	miR-132-3p	2013	up	cell lines and tissue	qRT-PCR	[62]	8
miR-224	miR-224-5p	2013	up	cell lines and tissue	qRT-PCR	[62]	13
let-7g	let-7g-5p	2013	up	cell lines and tissue	qRT-PCR	[62]	17
miR-125b	miR-125b-5p	2008	down	Tissue	qRT-PCR	[63]	33
miR-137	miR-137	2008	down	Tissue	qRT-PCR	[63]	17
miR-451	miR-451a	2009	down	cell lines and tissue	qRT-PCR	[64]	4
miR-143	miR-143-3p	2011	down	Tissue	qRT-PCR	[65]	15
miR-145	miR-145-5p	2011	down	Tissue	qRT-PCR	[65]	36
miR-215	miR-215	2012	down	tissue	TLDA	[57]	1
miR-200c	miR-200c-3p	2013	down	tissue	qRT-PCR	[31]	13
miR-320a	miR-320a	2013	down	cell lines and tissue	qRT-PCR	[62]	15
miR-124	miR-124-3p	2014	down	cell lines and tissue	qRT-PCR	[66]	51
miR-16	miR-16-5p	2012	NA	tissue	qRT-PCR	[67]	7
miR-590-5p	miR-590-5p	2012	NA	tissue	qRT-PCR	[67]	14
miR-153	miR-153	2012	NA	tissue	qRT-PCR	[67]	1
miR-519c-3p	miR-519c-3p	2012	NA	tissue	qRT-PCR	[67]	1
miR-561	miR-561-3p	2012	NA	tissue	qRT-PCR	[67]	15

Abbreviations: NA: not available; qRT-PCR: quantitative real-time polymerase chain reaction; TLDA: TaqMan Low Density Array.

We calculated the NOD values of the 30 collected miRNAs based on human miRNA–mRNA regulatory network and found that all of them had independent regulatory power (NOD > 0). Twenty-one (70%) of the miRNAs showed high NOD values (≥ 5), which confirmed the hypotheses that miRNAs with larger NOD values are more likely to be related to the changes in biological systems and could be candidates for putative biomarker screening [11].

We further summarized the miRNAs based on their expression levels and conducted gene ontology (GO) analysis for up- and down-regulated miRNAs separately. As shown in Supplementary Tables S1 and S2, respectively, the enriched GO terms of up- and down-regulated miRNAs were approximately the same at the cellular component (CC) and molecular function (MF) level. However, at the biological process (BP) level, the up-regulated miRNAs tended to play functional roles in biosynthetic and cellular biosynthetic processes, whereas the down-regulated miRNAs were always related to metabolic processes and cell death.

### Candidate miRNA biomarkers for predicting response to preoperative chemoradiotherapy

**Figure 2:**
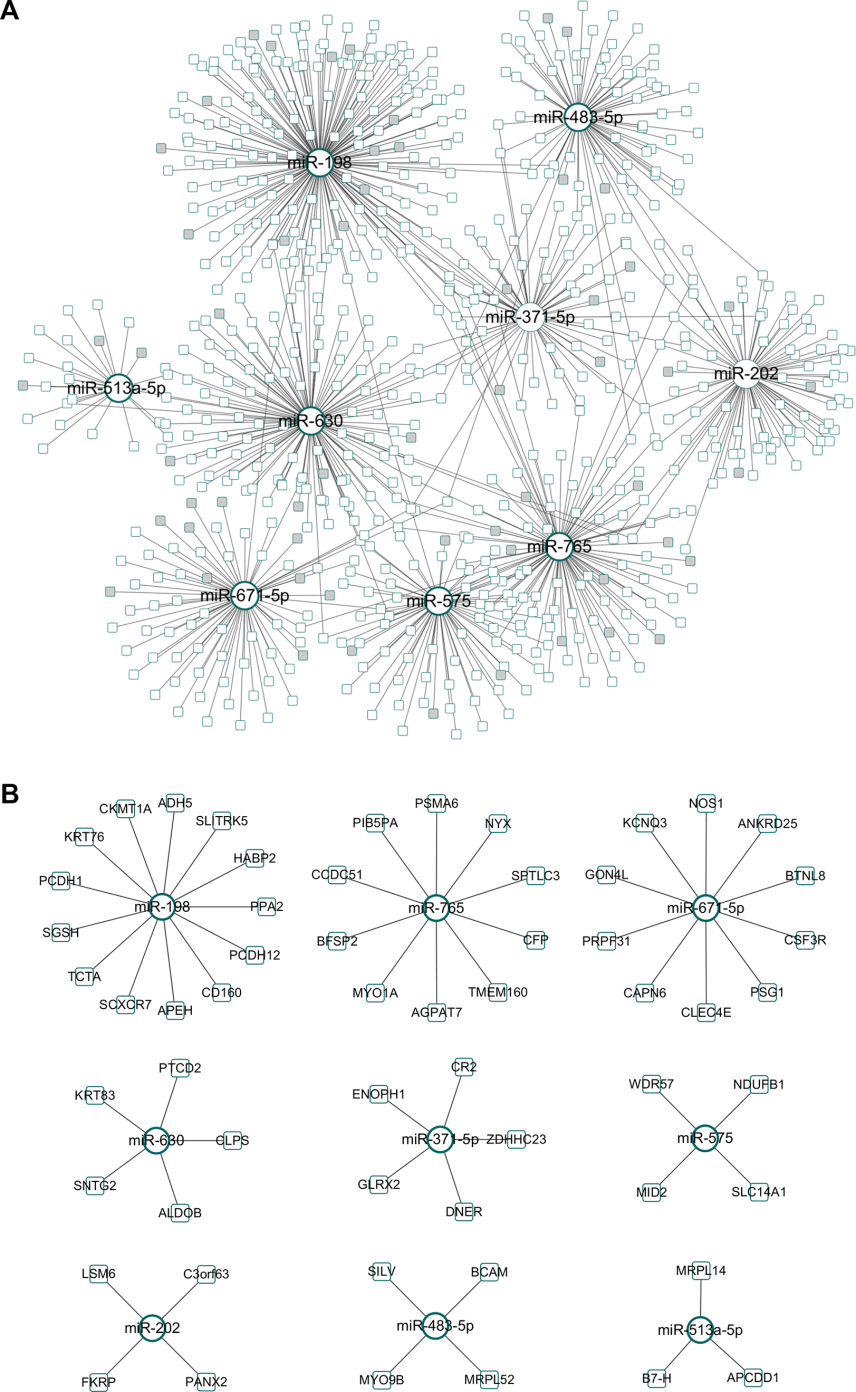
Identified miRNA biomarkers and their regulatory network (**A**) CRC chemoradiotherapy specific miRNA–mRNA network. The genes exclusively regulated by a specific miRNA were marked in grey. (**B**) Identified biomarker miRNAs and their unique target genes.

Forty-six differentially expressed (DE) miRNAs were obtained from the selected miRNA expression profile. Based on the POMA model, nine miRNAs with significantly high NOD values (*p-value* < 0.05, Wilcoxon signed-rank test), that is, miR-198, miR-765, miR-671-5p, miR-630, miR-371-5p, miR-575, miR-202, miR-483-5p and miR-513a-5p, were identified as candidate biomarkers for predicting CRC response to preoperative chemoradiotherapy. These nine outliers and their 713 target genes constituted the CRC chemoradiotherapy specific miRNA–mRNA network with 768 regulatory pairs (Figure 2A). The unique targets for each miRNA are highlighted in Figure 2B. The details of the candidate biomarker miRNAs can be found in Table 2.

**Table 2:** Candidate miRNA biomarkers identified by POMA model

miRNA ID	*P*-value (responders versus non-responders)	Number of targets	NOD value (*P*-value)
miR-198	6.80 E-03	174	13 (2.98E-08)
miR-765	1.39 E-05	98	10 (1.19E-07)
miR-671-5p	5.96 E-04	68	10 (1.19E-07)
miR-630	9.69 E-04	121	5 (4.37 E-03)
miR-371-5p	2.11 E-03	63	5 (4.37 E-03)
miR-575	4.07 E-03	65	4 (1.75 E-02)
miR-202	1.40 E-03	82	4 (1.75 E-02)
miR-483-5p	1.44 E-04	74	4 (1.75 E-02)
miR-513a-5p	3.01E-04	23	3 (4.78 E-02)

Note: *p*- values in Column 2 and 4 were calculated by Student's *t* test and Wilcoxon signed-rank test, respectively. Abbreviations: POMA: Pipeline of Outlier MicroRNA Analysis.

**Figure 3:**
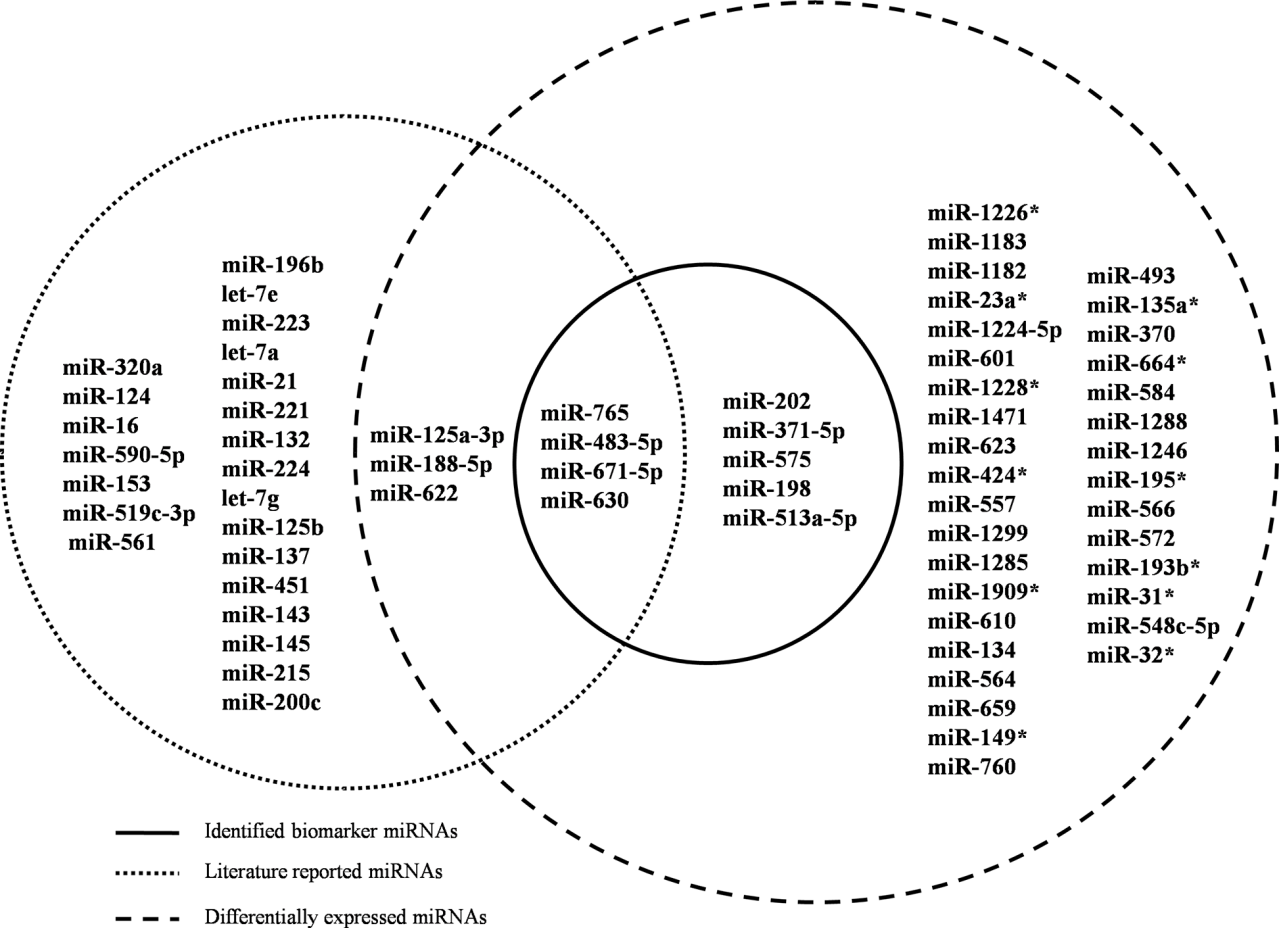
Venn diagram for literature-reported chemoradiotherapy associated miRNAs, DE miRNAs and identified miRNA biomarkers Dashed circles on the left and right represent literature-reported miRNAs and DE miRNAs, respectively. Nine miRNAs in the solid circle are candidate biomarkers identified by POMA model. Abbreviations: DE: differentially expressed; POMA: Pipeline of Outlier MicroRNA Analysis.

A literature search was performed on the nine identified miRNA biomarkers to validate their relevance to the regulation of CRC chemoradiotherapy. miR-765, miR-671-5p, miR-483-5p and miR-630 are reported to be powerful enough to predict the response of CRC to preoperative radiotherapy (Figure 3) [14]. miR-371 can modulate the Wnt/β-catenin-signaling pathway transactivated by β-catenin/LEF1 [15], which is an important pathway in regulating chemoradiation. miR-202 is involved in colon cancer pathways and associated with cyto- or chemokine expression after radiotherapy [16]. miR-198 is a tumor suppressor that inhibits proliferation, induces apoptosis, and represses tumor growth as well as metastasis in many cancers, including CRC [17]. Although the remaining two miRNAs are not reported to have direct relationships with CRC or to affect radiotherapy, they play crucial roles in other cancers. For example, miR-575 contributes to non-small cell lung cancer cell growth and invasion [18], whereas miR-513 combines with 12 other miRNAs as a 13-miRNA signature that participates in regulating tumorigenesis [19].

### Integrative bioinformatics analysis of identified miRNA biomarkers

We supposed that if the identified biomarker miRNAs could predict the CRC response to preoperative chemoradiotherapy, the genes regulated by them should also be involved in CRC initiation and progression. Here miRNA targets were extracted from human miRNA–mRNA networks, in which both experimentally validated and computationally predicted miRNA–mRNA pairs were integrated (Supplementary Table S3). We performed GO and pathway enrichment analyses on targets of identified biomarker miRNAs to explore the function and pathogenesis of these candidate biomarkers.

**Figure 4:**
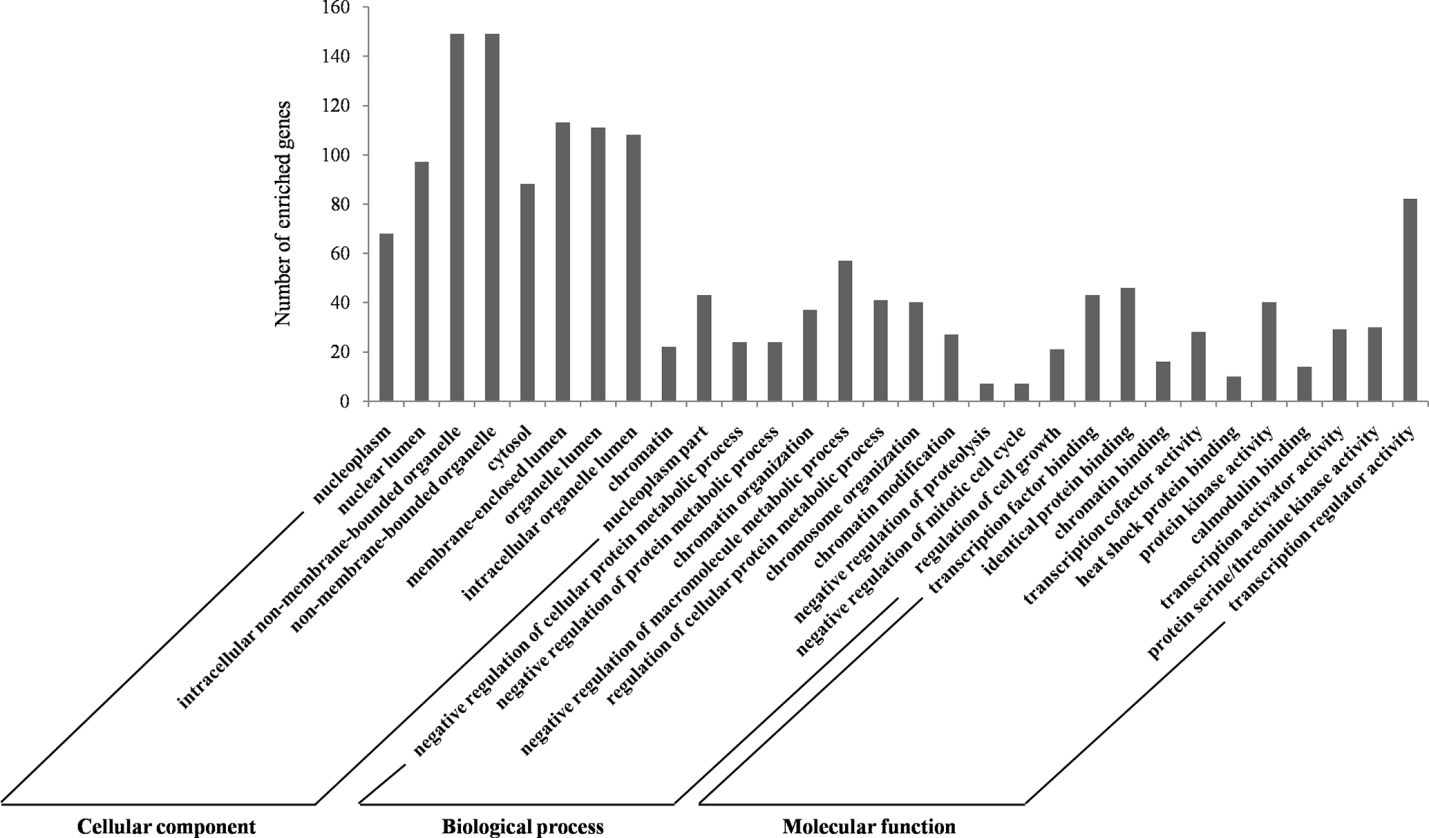
GO annotation of genes regulated by identified biomarker miRNAs The targeted genes by identified biomarker miRNAs were annotated by DAVID tools at three levels, including molecular function, cellular component, and biological process. The top 10 significantly enriched items for each domain are shown. Abbreviations: DAVID, Database for Annotation, Visualization, and Integrated Discovery; GO: Gene Ontology.

We carried out the GO analysis using the Database for Annotation, Visualization, and Integrated Discovery (DAVID) at three different GO levels: MF, CC and BP. Figure 4 illustrates the top 10 items that were significantly enriched by target genes for each of the above GO levels. The enriched GO terms in MF mainly included transcription factor binding, transcription cofactor activity, transcription activator activity and transcription regulator activity, which were involved in the processes of CRC chemoradiotherapy [20, 21]. CC items were associated with the hallmarks of a cell: nucleoplasm, organelle, cytosol and chromatin, which were critical areas with a major influence on radiation sensitivity [22, 23]. Most GO BP items converged on the regulation processes such as regulating the protein metabolic process and cell cycle [24, 25]. The GO annotation results supported the correlation between our identified miRNAs and CRC chemoradiotherapy.

**Figure 5:**
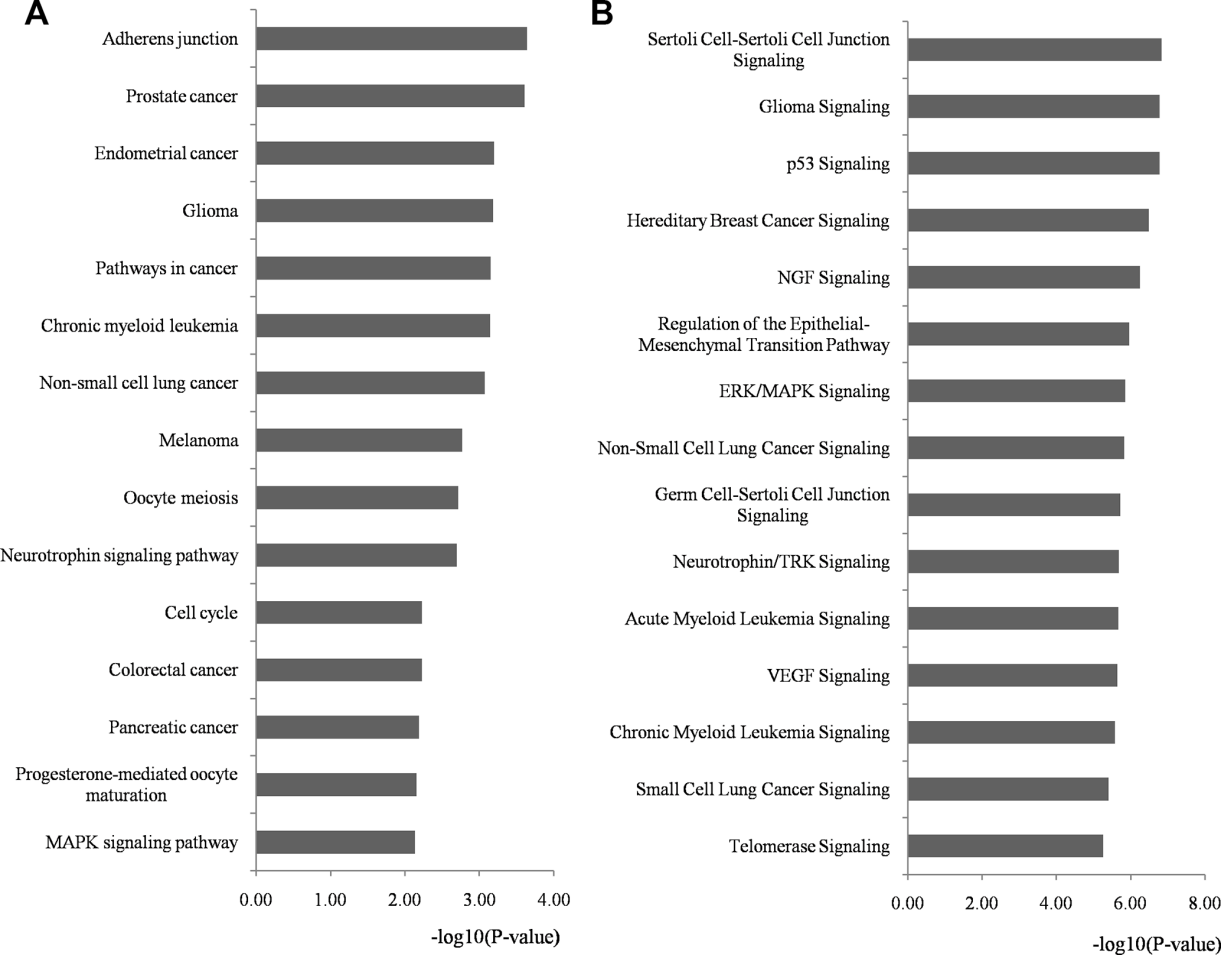
Pathway enrichment analysis for genes regulated by identified miRNA biomarkers Genes regulated by identified miRNA biomarkers were retrieved and enriched in KEGG and IPA pathways with DAVID and IPA program, respectively. The statistical significance level (*p-value*) was negative 10-based log transformed. (**A**) The top 15 significantly enriched KEGG pathways (*p-value* < 0.01). (**B**) The top 15 significantly enriched IPA pathways (*p-value* < 0.01). Abbreviations: DAVID, Database for Annotation, Visualization, and Integrated Discovery; IPA, Ingenuity Pathway Analysis; KEGG, Kyoto Encyclopedia of Genes and Genomes.

KEGG (Kyoto Encyclopedia of Genes and Genomes) and IPA (Ingenuity Pathway Analysis) pathway analyses were performed on the whole targets of miRNA biomarkers using DAVID and IPA program, respectively (Supplementary Tables S4 and S5, respectively). The top 15 significantly enriched pathways of both KEGG and IPA are outlined in Figure 5A and 5B, respectively and details are listed in Supplementary Tables S6 and S7, respectively. We identified several novel CRC-associated pathways from the top 15 enriched KEGG terms, namely pathways in cancer, cell cycle and mitogen-activated protein kinase (MAPK) signaling, which were related to response to chemoradiotherapy. The top enriched IPA terms also revealed some pathways that were related to the response to chemoradiotherapy such as p53, extracellular signal-regulated kinase (ERK)/MAPK, vascular endothelial growth factor (VEGF) and telomerase signaling. [26–33].

**Figure 6:**
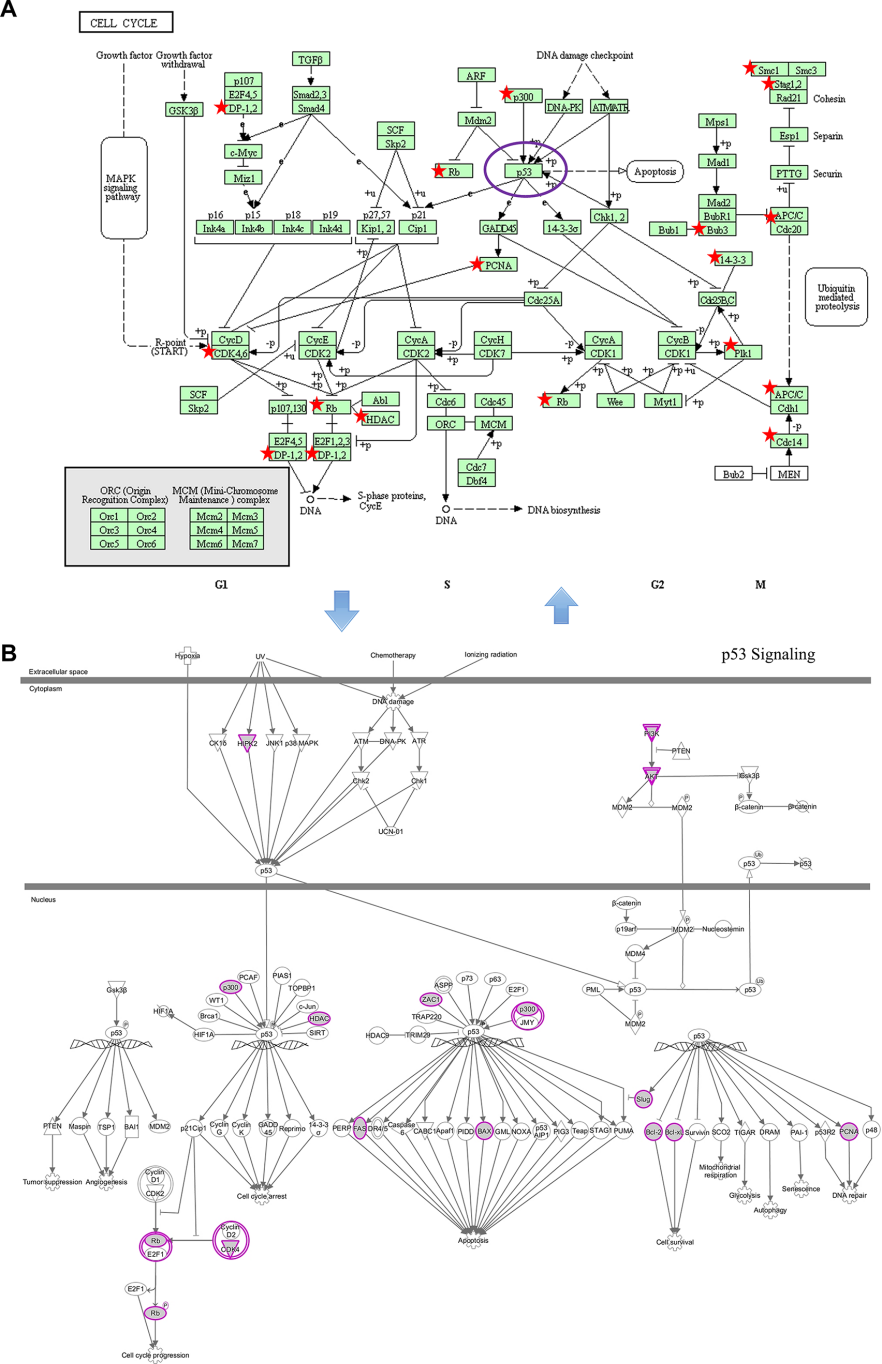
Examples of significantly enriched pathways for KEGG and IPA pathways (**A**) Cell cycle pathway (from KEGG). (**B**) p53 signaling pathway (from IPA). Objects with pentagrams in sub- and purple circles in sub- were acting locus by mapped genes. Abbreviations: IPA, Ingenuity Pathway Analysis; KEGG, Kyoto Encyclopedia of Genes and Genomes.

Many studies were critically reviewed with respect to the role of the cell cycle in the cellular response to radiation [27]. The mechanisms that are responsible for regulating ionizing radiation are inextricably linked to the checkpoint control system involved mainly in cell cycle arrest and DNA damage repair [28]. Checkpoint kinase signaling inhibitors may provide potential therapeutic applications in radiation oncology [29]. The well-studied p53 signaling is recognized as a key pathway influencing cellular oncogenesis, genomic stability, DNA damage repair, and apoptosis [34]. Mediated by miRNA targets, p53 signaling alterations could modify the intrinsic radiosensitivity of normal and tumor cells through the checkpoint control system [35]. It is particularly interesting that direct connections exist between the cell cycle pathway and p53 signaling. So, we reconstructed the two pathways from KEGG and IPA, respectively. The cell cycle and p53 signaling pathways were fitted well by the regulated genes of the nine miRNAs (Figure 6A, 6B). We found that five genes (EP300, HDAC1, PCNA, RB1 and CDK4) participated in the p53 and cell cycle signaling pathways, and it was not difficult to explain their correlations. p53 is one of the core parts of the cell cycle pathway, therefore, the two pathways complement each other to influence radiation sensitivity.

Pathways in cancer consist of many well-known signaling pathways, such as transforming growth factor (TGF)-β, VEGF, MAPK, Wnt and p53, which play essential roles in cell apoptosis, proliferation, differentiation, invasion and metastasis (Supplementary Figure S1) [26]. VEGF receptor signaling (Supplementary Figure S2), one of the above pathways in cancer, stimulates proliferation, promotes migration, and functions mainly in vasculogenesis, angiogenesis, endothelial integrity and survival [36]. VEGF signaling is activated by oncogenes, tumor suppressor genes, cytokines, and especially hypoxia. This leads to endothelial cell migration, neovascularization to support tumor growth, and creation of areas of hypoxia, which then cause tumor aggressiveness, poor survival and local treatment failure in CRC [32]. Anti-VEGF treatment with particular synthetic antisense antagomirs may significantly improve the effect of radiotherapy by normalizing the tumor vasculature, remodeling tumor vessels, improving vessel perfusion, and promoting oxygenation [33]. Accumulating evidence has indicated that MAPK signaling (Supplementary Figure S3) plays a pivotal part in CRC development, including cell proliferation, differentiation and apoptosis [30]. It is implicated that deregulated MAPK signaling has a high correlation with colorectal carcinogenesis. Suppression of the MAPK signaling pathway may provide a potential therapeutic approach to enhance the sensitivity of radiotherapy for human CRC [37].

All the above findings support the relationship between enriched pathways and radiation response. The evidence from GO and pathway enrichment analyses corroborates the reliability of the results in our study.

### Experimental validation of the identified biomarker miRNAs

To verify the bioinformatics model for predicting the response to preoperative chemoradiotherapy, the nine candidates identified were chosen for further quantitative PCR (q-PCR). It was difficult to obtain fresh CRC specimens, therefore, we chose a feasible but not perfect method to accomplish the validation in cell lines. HCT116 cells are recognized as a radiosensitive CRC cell line, whereas HT-29 cells are insensitive [38]. Thus, we established a cell model for CRC patients with radiotherapy in HCT116 and HT-29 cell lines.

**Figure 7:**
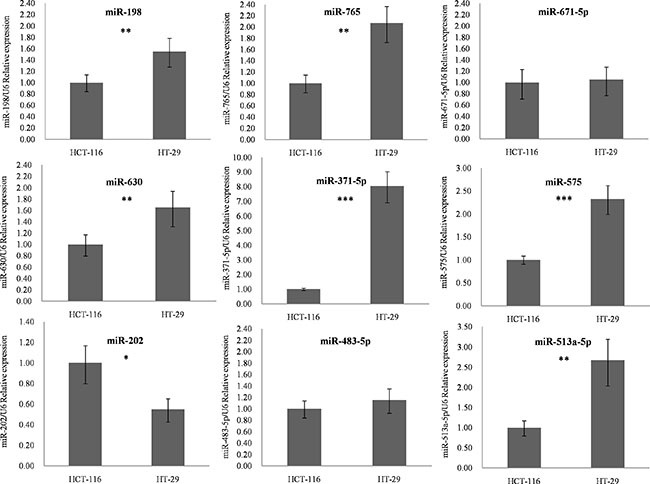
q-PCR results for identified miRNA biomarkers The nine candidates identified by our method were chosen to detect their outlier activity in colorectal cancer cell lines using q-PCR. Expression of these miRNAs was normalized against U6 snRNA expression. The statistical significance of differences between HCT116 and HT-29 cells was calculated using Student's *t* test. *p-value < 0.05, **p-value < 0.01, ***p-value < 0.001. Abbreviations: q-PCR, quantitative polymerase chain reaction.

As shown in Figure 7 and Supplementary Table S8, seven of the nine (77.8%) identified miRNAs were confirmed to have significant expression differences between the two cell lines (*p-value* < 0.05). miR-198, miR-765, miR-630, miR-371-5p, miR-575 and miR-513a-5p were overexpressed in HT-29 cells, whereas expression of miR-202 was reduced in the radio-insensitive CRC cell line. miR-671-5p and miR-483-5p did not show significant differences; however, they were still associated with CRC radiotherapy according to the study by Della et al. [14]. Clinical verification is therefore necessary.

**Figure 8:**
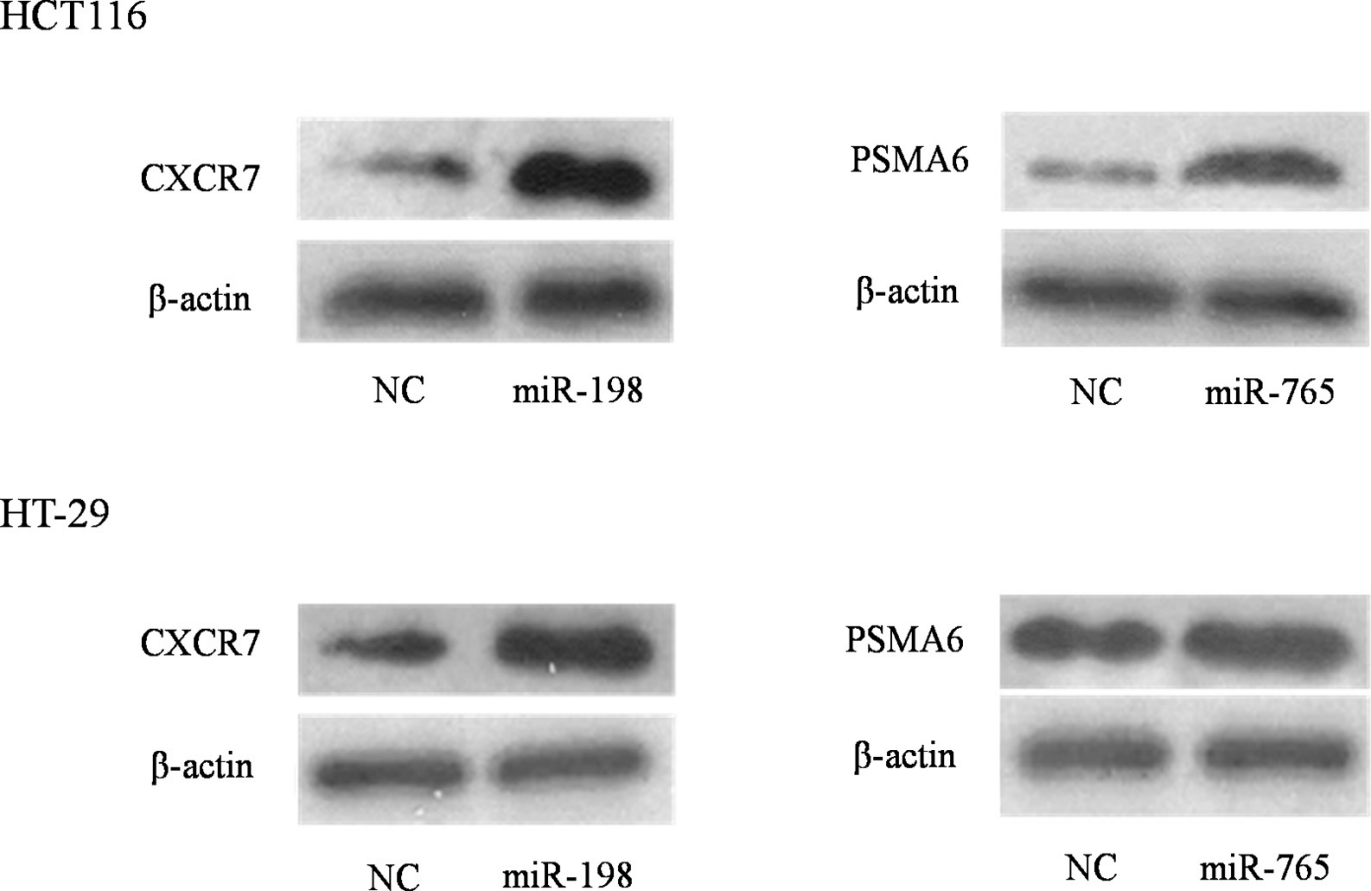
Experimental spot checking the unique target genes of identified biomarker miRNAs in colorectal cancer cell lines HCT116 and HT-29 cell lines were transfected with either miRNA or negative control mimics. Western blot analysis revealed that, upon transfection of miRNA mimics, their target proteins were altered (increased or decreased) significantly in HCT-116 while not in HT-29 cells.

In the POMA model, we paid more attention to the independent regulatory sites in miRNA–mRNA network because these positions were frail and changes in them could induce disorders at the systemic level. Thus, we randomly chose one uniquely targeted gene of miR-198 and miR-765 to validate their relationship to the miRNAs *in vitro*. HCT116 and HT-29 cell lines were transfected with the two miRNA or control mimics. Western blotting revealed that CXCR7 (*p-value* = 0.046) and PSMA6 (*p-value* = 0.0168) were altered significantly in HCT116 cells, which was in accordance with our basic hypothesis (Figure 8). The above alterations were not found in the HT-29 cell line. One reason could be that miRNAs more easily changed their target genes in the radiosensitive CRC cell line. Further investigations are needed to explore the mechanism.

For better understanding of the clinical significance of CXCR7 and PSMA6 genes in CRC tumors responsive to preoperative radiotherapy, we downloaded the gene expression profile GSE3493 [39] from NCBI GEO datasets. We analyzed differences in expression of the two genes between responders and non-responders to preoperative radiotherapy. The *p*- values calculated by Student's *t* test were 0.2739 and 0.3211 for CXCR7 and PSMA6, respectively. However, considering the individual differences among different patients, we further analyzed the expression level of these two genes based on single samples in the dataset. The two genes tended to be differentially expressed between radiotherapy responders and non-responders with |fold-change| ≥ 2, which indicated their clinical potential for personalized CRC treatment (Supplementary Table S9). Clinical verification is needed in the future when sufficient specimens are available for experimental research.

## DISCUSSION

miRNAs are stable in tissues and blood and are involved in radiation response, which may provide predictive and prognostic biomarkers in cancer [40]. The potential mechanism that underlies miRNA involvement in tumor radiation response converges on regulating DNA damage response, hypoxic tumor microenvironment, cancer stem cells and survival pathway changes [41]. However, the promising miRNAs as well as the specific mechanism that is responsible for the radiation response are still poorly understood.

In this study, we applied a functional miRNA prediction model to identify biomarkers for the CRC response to preoperative radiotherapy. The model considered the topological characteristic of miRNAs in miRNA–mRNA network and eliminated the inter-dataset inconsistency. We thought that the independent regulatory sites in the networks were frailer than synergistic ones, which may contribute to disorder at the systemic level.

We characterized the miRNAs with the potential to predict the CRC response to preoperative chemoradiotherapy and explored their topological features in miRNA–mRNA networks. Consistent with our foundational hypothesis, most of the reported biomarkers have independent regulatory powers (NOD ≥ 5), which provided a solid foundation for our intensive study.

Based on the findings above, we retrieved 46 DE miRNAs from the miRNA expression dataset. miR-125a-3p, miR-188-5p, miR-622, miR-765, miR-483-5p, miR-671-5p and miR-630 are associated with CRC chemoradiotherapy. After POMA filtration, miR-198, miR-765, miR-671-5p, miR-630, miR-371-5p, miR-575, miR-202, miR-483-5p and miR-513a-5p were identified as candidate biomarkers. Four of them were confirmed by published studies, which demonstrated the reliability of our predictive results.

We performed functional enrichment analysis on the whole target genes of identified miRNA biomarkers. Most enriched GO terms were significantly associated with transcription activity at the MF level, regulation processes at BP level and the basic cell structural at CC level, which agreed well with the regulatory concepts of miRNAs. We then performed KEGG and IPA pathway analysis to check the potential pathways involved in the radiation response of the predicted miRNA biomarkers, along with their target genes. We identified several novel CRC-associated pathways from the top 15 enriched KEGG terms, namely pathways in cancer, cell cycle and MAPK signaling, which correlated well with radiation response according to the text mining analysis. Meanwhile, the top enriched IPA terms also revealed some pathways that were related to the response to chemoradiotherapy such as p53, ERK/MAPK, VEGF and telomerase signaling. It is worth noting that the enriched pathways had close internal connections among each other. We reconstructed the significant cell cycle–p53 signaling pathway, and found that five genes (EP300, HDAC1, PCNA, RB1 and CDK4) participated in both pathways. The remaining pathways also have potential values for further investigation. The enriched pathways provided insight into the pathogenesis of identified miRNA biomarkers in the radiation response, which could help bring effective therapeutic strategies for tumor cell radiosensitization.

Biological experiments are necessary to validate the accuracy of our predictive results. We found that seven of the nine candidates were significantly differentially expressed between radiosensitive and insensitive CRC cell lines by qRT-PCR (*p <* 0.05). Although miR-671-5p and miR-483-5p did not show differential expression, they are reported to be associated with the efficacy of chemoradiotherapy in CRC [14], which suggests their potential for predicting the response to preoperative chemoradiotherapy.

miRNAs with strong independent regulatory power are more likely to be potential biomarkers in human cancer [11], thus, we randomly selected one unique target gene regulated by miR-198 and miR-765 for western blot analysis. The two proteins of miR-765 and miR-198 were altered significantly upon transfection of miRNA mimics into HCT116 cells, which was in accordance with our basic hypothesis. The alterations were not obvious in HT-29 cells and further experiments are therefore needed to explore the reason.

From the perspective of systems biology, single molecules are unlikely to dictate the disease evolutionary process at the systems level. Cancer is a complex disease, therefore, combined miRNAs or miRNA modules will help investigate and explain the internal mechanism as well as the external influence of cancer development. Future biomarker miRNA identification for the chemoradiotherapy response in CRC should be rooted in network paradigms [42].

In this study, we applied an integrative approach to identify miRNA biomarkers for prediction of response of CRC to chemoradiotherapy and performed functional analysis and experimentally spot checking to verify them; however, there were still some limitations. First, the ideal experimental material should be CRC tissue samples, and the cell lines used here cannot fully reflect the miRNA alterations in CRC patients. However, we used cell lines because of the difficulty of obtaining clinical specimens. Second, we only selected two CRC cell lines; one of which was radiosensitive line and the other was insensitive. More cell lines including those with the moderate levels of radiation sensitivity should be studied. Third, due to the lack of publicly available data in this area, our study only selected one suitable miRNA expression dataset. In addition, our prediction model still needs to be improved to consider carefully the features and functions of miRNA targets.

In summary, we applied an integrative approach to identify miRNA biomarkers for predicting the response of CRC to chemoradiotherapy. Nine miRNAs were screened as candidate biomarkers. miR-198, miR-765, miR-630, miR-371-5p, miR-575, miR-202 and miR-513a-5p were validated by low-throughput experiments, which could be prioritized for further clinical practice.

## MATERIALS AND METHODS

### Data collection

We conducted a thorough search for CRC chemoradiotherapy associated miRNAs by manually mining citations in PubMed with the key words “miRNA or microRNA”, “colorectal cancer OR rectal cancer OR rectum cancer OR colorectal carcinoma OR rectal carcinoma”, “chemoradiotherapy OR radiotherapy OR radiation”. miRNAs verified experimentally or identified by predictive methods were collected. The details are summarized in Table 1.

The miRNA expression profile (GSE29298) was retrieved from the public database NCBI GEO [43]. The dataset contained nine samples from responders and 29 from non-responders to preoperative radiotherapy in rectal cancer [14]. Normalized miRNA data were downloaded directly for further analysis and this profile consisted of the expression information of 851 miRNAs (Supplementary Table S10). We extracted DE miRNAs based on Student's *t* test in the Limma R package [44]. *p-value* < 0.05 and |fold-change| > 2 were chosen as cut-off criteria.

### Identification of miRNA biomarkers for efficacy of chemoradiotherapy in CRC

We previously developed a prediction model (termed POMA) to identify candidate miRNA biomarkers from the miRNA regulatory network [11, 45, 46]. NOD, which is the number of genes exclusively targeted by certain miRNAs (also known as unique target genes), was defined in POMA to measure the independent regulatory power of an individual miRNA. Based on the hypotheses that miRNA activity is reflected by deregulated expression of its target genes, and miRNAs with larger NOD values are more likely to be biomarkers, POMA has been successfully conducted in prostate cancer [11, 12], clear cell renal cell carcinoma [46], sepsis [45] and acute myeloid leukemia [13]. We extended application of the POMA model to evaluation of CRC chemoradiotherapy associated miRNAs, and applied it to identifying novel miRNA biomarkers for predicting the response of CRC to preoperative chemoradiotherapy.

Based on POMA, the procedure of our study could be described as follows: (1) constructing human miRNA–mRNA regulatory network by integrating possible human miRNA–mRNA target pairs from four experimentally validated databases (miRecords [47], Tarbase [48], miR2Disease [49] and miRTarbase [50]) and three computationally predicted databases (HOCTAR [51], ExprTargetDB [52] and starBase [53]); (2) detecting DE miRNAs associated with efficacy of chemoradiotherapy in CRC from selected miRNA expression profiles (GSE29298) by statistical methods; (3) mapping DE miRNAs onto human miRNA–mRNA network and calculating the NOD values for each miRNA; and (4) identifying candidate miRNA biomarkers based on NOD distribution (*p-value* < 0.05, Wilcoxon signed-rank test).

### Functional enrichment analyses

We conducted GO analysis for CRC chemoradiotherapy associated miRNAs and identified biomarker miRNAs reported in the literature. Pathway enrichment analysis was performed to investigate the correlation between the targets of identified biomarker miRNAs and CRC chemoradiotherapy. miRNA targets were retrieved from the constructed miRNA–mRNA networks. The online tool DAVID [54] was used for GO and KEGG pathway analysis [55]. The IPA program was also applied to pathway enrichment analyses. The top 15 significantly enriched pathways (*p-value* < 0.01) were selected and confirmed for their correlation with CRC chemoradiotherapy by a literature search in PubMed.

### Cell culture

We used two human CRC cell lines: one radiosensitive (HCT116) and one insensitive (HT-29). The cell lines were purchased from the Shanghai Cell Biology, University of the Chinese Academy of Sciences and cultured in RPMI-1640 medium (Gibco) with 10% fetal bovine serum (Gibco), 1% streptomycin–penicillin (Invitrogen), and 1% L-glutamine (Invitrogen). All cell lines were incubated in a humidified atmosphere containing 5% CO_2_ at 37°C.

### q-PCR

Real-time q-PCR was used to investigate the expression of selected miRNA biomarkers in radiosensitive HCT116 cells and insensitive HT-29 cells. Total RNA was extracted from cultured cells using TRIzol reagent (Invitrogen). miRNAs were analyzed by a two-step q-PCR using the PrimeScript RT reagent kit (TaKaRa, Dalian, China). The first step included total RNA polyadenylation and reverse transcription, followed by q-PCR. miRNA stem-loop primers were purchased from Guangzhou Ribo BioCompany. Real-time q-PCRs were performed using an Applied Biosystems 7500 Fast Real-time PCR system with an SYBR Green Premix Ex Taq kit (TaKaRa, China). Every step was performed in triplicate. All q-PCR values of each miRNA were normalized against U6 snRNA. The relative expressions of five miRNAs were calculated using the 2^−ΔΔCt^ method [56].

### Transfection of miRNA mimics

For transient transfection, cells were transfected using Lipofectamine 3000 (Invitrogen) at 50% confluence with miRNA or control mimics (Qiagen).

### Western blotting

Each group of cells was washed with PBS and lysed with an equal volume of RIPA buffer on ice. Equivalent amounts of proteins were resolved by SDS-PAGE and transferred to polyvinyl difluoride membranes (Millipore, Billerica, MA, USA). After blocking with 5% non-fat milk in TBST for 1 h at room temperature, the membranes were incubated with diluted primary antibodies against PSMA6 (Abcam, #ab109377), CXCR7 (Abcam, #ab138509) or β-actin (Cell Signaling Technology, #4970L) overnight at 4°C. Then, they were blotted for 1 h at room temperature with horseradish-peroxidase-conjugated secondary antibody, washed again in TBST, and developed in the ECL Plus Western Blotting Detection System.

### Statistical analysis

Statistical analysis was performed using R packages. Data were presented as mean ± standard deviation. The results were analyzed using ANOVA or Student's *t* test. Statistically significance for hypothesis test was set to *p-value* < 0.05 or < 0.01, where the latter was stricter.

## SUPPLEMENTARY MATERIALS FIGURES AND TABLES




