# Supplementary material for: Identification of biomarker microRNAs for predicting the response of colorectal cancer to neoadjuvant chemoradiotherapy based on microRNA regulatory network
Source: Oncotarget. 2016 Nov 26;8(2):2233–48. doi: 10.18632/oncotarget.13659 (PMC5356795; doi:10.18632/oncotarget.13659)
Supplement: Supplementary file 1 [file oncotarget-08-2233-s001.pdf]

Identification of biomarker microRNAs for predicting the response of colorectal cancer to neoadjuvant chemoradiotherapy based on microRNA regulatory network

Supplementary Materials

Supplementary Table S1: The top 10 significantly enriched gene ontology (GO) terms by targets of reported *up-regulated* CRC chemoradiotherapy associated miRNAs

GO terms	Number of enriched genes	*P*-value
**Cellular component**		
**0031974~membrane-enclosed lumen**	323	2.38E-23
**0031981~nuclear lumen**	267	1.50E-22
**0043233~organelle lumen**	313	1.06E-21
**0005654~nucleoplasm**	185	1.27E-21
**0070013~intracellular organelle lumen**	307	1.73E-21
**0044451~nucleoplasm part**	120	6.32E-15
0043232~intracellular non-membrane-bounded organelle	382	2.02E-14
0043228~non-membrane-bounded organelle	382	2.02E-14
**0005829~cytosol**	217	4.22E-12
0012505~endomembrane system	140	1.06E-10
**Biological process**		
**0010605~negative regulation of macromolecule metabolic process**	173	2.64E-21
0010629~negative regulation of gene expression	125	2.40E-17
0009890~negative regulation of biosynthetic process	136	4.12E-17
0031327~negative regulation of cellular biosynthetic process	132	4.82E-16
0010558~negative regulation of macromolecule biosynthetic process	129	8.21E-16
0010604~positive regulation of macromolecule metabolic process	176	4.31E-15
0032268~regulation of cellular protein metabolic process	112	7.14E-14
0010557~positive regulation of macromolecule biosynthetic process	138	7.54E-13
0009891~positive regulation of biosynthetic process	144	9.80E-13
0031328~positive regulation of cellular biosynthetic process	142	1.37E-12
**Molecular function**		
**0008134~transcription factor binding**	117	6.50E-14
**0030528~transcription regulator activity**	259	5.14E-13
0003682~chromatin binding	48	2.78E-11
**0016563~transcription activator activity**	87	6.44E-9
0019899~enzyme binding	104	7.34E-9
**0016564~transcription repressor activity**	71	1.71E-8
**0003712~transcription cofactor activity**	75	2.49E-7
0019838~growth factor binding	32	2.92E-7
0042802~identical protein binding	114	6.13E-7
**0003700~transcription factor activity**	159	9.31E-7

Note: The GO terms in bold means they are also enriched by targets of down-regulated miRNAs (see Supplementary Table S2).

Supplementary Table S2: The top 10 significantly enriched gene ontology (GO) terms by targets of reported *down-regulated* CRC chemoradiotherapy associated miRNAs

GO terms	Number of enriched genes	*P*-value
**Cellular component**		
**0005654~nucleoplasm**	147	2.85E-19
**0031981~nuclear lumen**	204	5.42E-18
**0031974~membrane-enclosed lumen**	244	6.70E-18
**0070013~intracellular organelle lumen**	236	9.50E-18
**0043233~organelle lumen**	239	1.91E-17
**0005829~cytosol**	170	5.07E-11
**0044451~nucleoplasm part**	89	9.71E-11
0005667~transcription factor complex	42	3.90E-8
0005794~Golgi apparatus	113	8.38E-8
0000267~cell fraction	133	1.18E-7
**Biological process**		
0006793~phosphorus metabolic process	155	3.06E-15
0006796~phosphate metabolic process	155	3.06E-15
0042127~regulation of cell proliferation	133	4.23E-15
0016265~death	122	8.65E-14
0042981~regulation of apoptosis	131	1.19E-13
**0010605~negative regulation of macromolecule metabolic process**	122	2.33E-13
0043067~regulation of programmed cell death	131	2.54E-13
0008219~cell death	120	2.82E-13
0010941~regulation of cell death	131	3.33E-13
0012501~programmed cell death	106	7.36E-13
**Molecular function**		
**0030528~transcription regulator activity**	199	1.61E-11
**0008134~transcription factor binding**	86	4.91E-10
0004672~protein kinase activity	93	9.09E-9
**0003700~transcription factor activity**	128	1.67E-7
**0016563~transcription activator activity**	66	3.06E-7
0004674~protein serine/threonine kinase activity	68	3.91E-7
**0016564~transcription repressor activity**	54	6.65E-7
**0003712~transcription cofactor activity**	59	1.04E-6
0004721~phosphoprotein phosphatase activity	33	5.25E-6
0019904~protein domain specific binding	53	6.18E-6

Note: The GO terms in bold means they are also enriched by targets of up-regulated miRNAs (see Supplementary Table S1).

Supplementary Table S3: The miRNA-mRNA pairs in CRC specific miRNA-mRNA network.
See Supplementary_Table_S3

Supplementary Table S4: The significantly enriched (*p*-value < 0.01) KEGG pathways by targets of identified miRNA biomarkers

No.	Pathway term	*P*-value
1	hsa04520:Adherens junction	2.30E-04
2	hsa05215:Prostate cancer	2.47E-04
3	hsa05213:Endometrial cancer	6.39E-04
4	hsa05214:Glioma	6.59E-04
5	hsa05200:Pathways in cancer	7.09E-04
6	hsa05220:Chronic myeloid leukemia	7.14E-04
7	hsa05223:Non-small cell lung cancer	8.50E-04
8	hsa05218:Melanoma	1.71E-03
9	hsa04114:Oocyte meiosis	1.92E-03
10	hsa04722:Neurotrophin signaling pathway	2.02E-03
11	hsa04110:Cell cycle	5.90E-03
12	hsa05210:Colorectal cancer	5.95E-03
13	hsa05212:Pancreatic cancer	6.48E-03
14	hsa04914:Progesterone-mediated oocyte maturation	7.02E-03
15	hsa04010:MAPK signaling pathway	7.37E-03
16	hsa04150:mTOR signaling pathway	1.06E-02
17	hsa05216:Thyroid cancer	1.07E-02
18	hsa04910:Insulin signaling pathway	1.11E-02
19	hsa04360:Axon guidance	1.85E-02
20	hsa05221:Acute myeloid leukemia	1.88E-02
21	hsa04530:Tight junction	2.43E-02
22	hsa04310:Wnt signaling pathway	2.60E-02
23	hsa05412:Arrhythmogenic right ventricular cardiomyopathy (ARVC)	2.64E-02
24	hsa04510:Focal adhesion	2.79E-02
25	hsa04720:Long-term potentiation	4.08E-02
26	hsa05222:Small cell lung cancer	4.43E-02
27	hsa05211:Renal cell carcinoma	4.66E-02
28	hsa04810:Regulation of actin cytoskeleton	4.78E-02

Supplementary Table S5: The top 40 significantly enriched IPA pathways (*P*-value < 0.01) enriched by targets of identified miRNA biomarkers

No.	Pathway term	*P*-value
1	Sertoli Cell-Sertoli Cell Junction Signaling	1.48E-07
2	p53 Signaling	1.62E-07
3	Glioma Signaling	1.62E-07
4	Hereditary Breast Cancer Signaling	3.24E-07
5	NGF Signaling	5.62E-07
6	Regulation of the Epithelial-Mesenchymal Transition Pathway	1.07E-06
7	ERK/MAPK Signaling	1.38E-06
8	Non-Small Cell Lung Cancer Signaling	1.48E-06
9	Germ Cell-Sertoli Cell Junction Signaling	1.91E-06
10	Neurotrophin/TRK Signaling	2.09E-06
11	Acute Myeloid Leukemia Signaling	2.14E-06
12	VEGF Signaling	2.29E-06
13	Chronic Myeloid Leukemia Signaling	2.63E-06
14	Small Cell Lung Cancer Signaling	3.89E-06
15	Telomerase Signaling	5.50E-06
16	GM-CSF Signaling	6.03E-06
17	Molecular Mechanisms of Cancer	6.76E-06
18	Breast Cancer Regulation by Stathmin1	7.24E-06
19	Epithelial Adherens Junction Signaling	8.51E-06
20	Melanoma Signaling	8.51E-06
21	NRF2-mediated Oxidative Stress Response	1.07E-05
22	Production of Nitric Oxide and Reactive Oxygen Species in Macrophages	1.07E-05
23	Hepatic Fibrosis / Hepatic Stellate Cell Activation	1.38E-05
24	Ceramide Signaling	1.41E-05
25	Prostate Cancer Signaling	1.82E-05
26	Myc Mediated Apoptosis Signaling	2.09E-05
27	IGF-1 Signaling	2.14E-05
28	Melanocyte Development and Pigmentation Signaling	2.34E-05
29	Ephrin Receptor Signaling	2.40E-05
30	Axonal Guidance Signaling	3.02E-05
31	FAK Signaling	3.31E-05
32	Glioblastoma Multiforme Signaling	3.31E-05
33	FLT3 Signaling in Hematopoietic Progenitor Cells	3.47E-05
34	Ovarian Cancer Signaling	3.47E-05
35	14-3-3-mediated Signaling	3.89E-05
36	Actin Cytoskeleton Signaling	4.37E-05
37	Rac Signaling	4.57E-05
38	Role of p14/p19ARF in Tumor Suppression	4.90E-05
39	Endometrial Cancer Signaling	5.25E-05
40	Aldosterone Signaling in Epithelial Cells	5.50E-05

Supplementary Table S6: The details of top 15 significantly enriched KEGG pathways

Pathway term	*P*-value	Gene count	Enriched genes
hsa04520:Adherens junction	2.30 E-04	13	CSNK2A2,TJP1,EP300,PVRL1,FYN,PVRL3,MAPK3,RHOA,LEF1,SNAI2,CTNNA1,TCF7L1, CTNNA3
hsa05215:Prostate cancer	2.47 E-04	14	FGFR2,MAP2K1,PIK3CD,LEF1,IGF2,RB1,TCF7L1,EP300,BCL2,ARAF,MAPK3,PDGFD,PIK3R1, AKT2
hsa05213:Endometrial cancer	6.39 E-04	10	MAP2K1,ARAF,MAPK3,PIK3CD,LEF1,CTNNA1,TCF7L1,CTNNA3, PIK3R1, AKT2
hsa05214:Glioma	6.59E-04	11	MAP2K1,CAMK2G,ARAF,MAPK3,PIK3CD,CDK6,SHC1,RB1,CDK4,PIK3R1, AKT2
hsa05200:Pathways in cancer	7.09 E-04	30	FGFR2,TFG,BCL2L1, TCF7L1, TPM3, MAX, BCL2, RHOA, CSF3R, FAS, FGF1, CSF2RA, PIK3R1, AKT2, DVL3, MAP2K1, PIK3CD, LEF1, CDK6, RB1, CDK4, CTNNA1, STK4, CTNNA3, EP300, HDAC1, BAX, ARAF, MAPK3, WNT7A
hsa05220:Chronic myeloid leukemia	7.14 E-04	12	MAP2K1, HDAC1, ARAF, MAPK3, PIK3CD, CDK6, SHC1, RB1, BCL2L1, CDK4, PIK3R1, AKT2
hsa05223:Non-small cell lung cancer	8.50 E-04	10	MAP2K1,ARAF, MAPK3, PIK3CD, CDK6, RB1, CDK4, STK4, PIK3R1, AKT2
hsa05218:Melanoma	1.71E-03	11	MAP2K1, ARAF, MAPK3, PIK3CD, CDK6, RB1, PDGFD, CDK4, FGF1, PIK3R1, AKT2
hsa04114:Oocyte meiosis	1.92 E-03	14	PPP2R1A, PPP2R5B, MAP2K1, CAMK2G, IGF2, CDC27, PPP1CA, RPS6KA3, RPS6KA1, PLK1, MAPK3, PPP2CB, YWHAQ, SMC1A
hsa04722:Neurotrophin signaling pathway	2.02 E-03	15	MAP2K1, CAMK2G, PIK3CD, NTRK3, RPS6KA3, RPS6KA1, BAX, BCL2, MAP3K1, MAPK3, YWHAQ, RHOA, SHC1, PIK3R1, AKT2
hsa04110:Cell cycle	5.90 E-03	14	EP300,CDC14A,HDAC1,PLK1,YWHAQ, PCNA, CDK6, RB1, CDK4, SMC1A, CDC27, STAG2, BUB3, TFDP1
hsa05210:Colorectal cancer	5.9 E-03	11	DVL3, MAP2K1, BCL2, BAX, ARAF, MAPK3, PIK3CD, LEF1, TCF7L1, PIK3R1, AKT2
hsa05212:Pancreatic cancer	6.48 E-03	10	MAP2K1,ARAF,MAPK3,PIK3CD,CDK6,RB1,BCL2L1,CDK4, PIK3R1,AKT2
hsa04914:Progesterone-mediated oocyte maturation	7.02E-03	11	RPS6KA3, RPS6KA1, MAP2K1, PLK1, ARAF, MAPK3, PIK3CD, IGF2, CDC27, PIK3R1, AKT2
hsa04010:MAPK signaling pathway	7.37 E-03	23	FGFR2,CACNA2D1,MAP2K1,TAOK3,CACNB1, MAPKAPK3, NR4A1, STK4, MAX, RPS6KA3, MAP3K4, RPS6KA1, PAK2, MAP3K2, MAP3K1, MAPK3, CACNA1G, RRAS, FAS, STMN1, FGF1, PPP5C, AKT2

Supplementary Table S7: The details of top 15 significantly enriched IPA pathways

Pathway term	*P*-value	Gene count	Enriched genes
Sertoli Cell-Sertoli Cell Junction Signaling	1.48E-07	22	NOS1,SPTBN1,SPTBN2,EPN1,AKT2,RRAS,TJP1,TUBG1,PVRL3,MAP3K1,JAM2,CTNNA1,MAP3K4,TUBB,ACTA2,MAPK3,SPTB,PRKAG2,PVRL1,CLINT1,MAP2K1,MAP3K2
p53 Signaling	1.62E-07	16	AKT2,SNAI2,PIK3R1,PLAGL1,HDAC1,PIK3C2G,CDK4,BAX,FAS,BCL2,EP300,RB1,BCL2L1,PCNA,PIK3CD,HIPK2
Glioma Signaling	1.62E-07	16	AKT2,TFDP1,RRAS,PIK3R1,PIK3C2G,CDK4,CDK6,IDH1,SHC1,RB1,IGF2,MAPK3,PIK3CD,PDGFD,MAP2K1,CAMK2G
Hereditary Breast Cancer Signaling	3.24E-07	18	AKT2,ARID1A,DPF1,RRAS,PIK3R1,TUBG1,HDAC1,PIK3C2G,CDK6,CDK4,SMARCA4,EP300,HDAC6,RB1,SMARCA2,H2AFX,PIK3CD,RFC3
NGF Signaling	5.62E-07	16	AKT2,RRAS,PIK3R1,MAP3K1,RPS6KA3,PIK3C2G,BAX,MAP3K4,EP300,SHC1,RHOA,MAPK3,PIK3CD,RPS6KA1,MAP2K1,MAP3K2
Regulation of the Epithelial-Mesenchymal Transition Pathway	1.07E-06	21	AKT2,SNAI2,RRAS,PIK3R1,PIK3C2G,FGFR2,BCL9,TCF7L1,FGF1,PYGO2,WNT7A,ARAF,APH1A,MAPK3,RHOA,LEF1,DVL3,PIK3CD,PDGFD,MAP2K1,HMGA2
ERK/MAPK Signaling	1.38E-06	21	MYCN,FYN,RRAS,PPP1R3C,PIK3R1,PPP2R5B,PIK3C2G,EP300,YWHAQ,PPP2CB,SHC1,PPP2R1A,H3F3A/H3F3B,ARAF,MAPK3,PAK2,PRKAG2,PIK3CD,RPS6KA1,PPP1CA,MAP2K1
Non-Small Cell Lung Cancer Signaling	1.48E-06	12	STK4,RB1,AKT2,TFDP1,RRAS,PIK3R1,MAPK3,CDK6,CDK4,PIK3C2G,PIK3CD,MAP2K1
Germ Cell-Sertoli Cell Junction Signaling	1.91E-06	19	EPN1,RRAS,TJP1,PIK3R1,TUBG1,PVRL3,MAP3K1,PIK3C2G,CTNNA1,MAP3K4,TUBB,ACTA2,MAPK3,RHOA,PAK2,PIK3CD,CLINT1,MAP2K1,MAP3K2
Neurotrophin/TRK Signaling	2.09E-06	12	SHC1,SPRY1,RRAS,NTRK3,SPRY2,PIK3R1,MAPK3,PIK3C2G,PIK3CD,RPS6KA1,MAP2K1,EP300
Acute Myeloid Leukemia Signaling	2.14E-06	13	CSF3R,AKT2,ARAF,CSF2RA,RRAS,MAPK3,PIK3R1,PIK3C2G,LEF1,PIK3CD,TCF7L1,MAP2K1,IDH1
VEGF Signaling	2.29E-06	14	SH2D2A,SHC1,BCL2L1,AKT2,ACTA2,RRAS,PIK3R1,MAPK3,PIK3C2G,EIF2B3,PIK3CD,KDR,MAP2K1,BCL2
Chronic Myeloid Leukemia Signaling	2.63E-06	14	HDAC6,BCL2L1,RB1,AKT2,TFDP1,RRAS,PIK3R1,MAPK3,HDAC1,CDK6,CDK4,PIK3C2G,PIK3CD,MAP2K1
Small Cell Lung Cancer Signaling	3.89E-06	12	NOS1,BCL2L1,RB1,AKT2,MAX,TFDP1,PIK3R1,CDK6,CDK4,PIK3C2G,PIK3CD,BCL2
Telomerase Signaling	5. 50E-06	14	HDAC6,RB1,SHC1,PPP2CB,PPP2R1A,AKT2,RRAS,MAPK3,PIK3R1,HDAC1,PPP2R5B,PIK3C2G,PIK3CD,MAP2K1

Supplementary Table S8: The statistical significance (*p*-value) of identified miRNA biomarkers between HCT116 and HT-29 group

miRNA ID	*p*-value
miR-198	0.007
miR-765	0.002
miR-671-5p	0.7551
miR-630	0.0086
miR-371-5p	0.000024
miR-575	0.00062
miR-202	0.0139
miR-483-5p	0.3332
miR-513a-5p	0.00219

Note: The statistical significance (*p*-value) was calculated using the Student's *t*-test.

Supplementary Table S9: Fold-changes of CXCR7 and PSMA6 expression between responders and non-responders to preoperative radiotherapy

Gene symbol	Treatment	Control	Fold-change
CXCR7	GSM79068	GSM79084	16.35
	GSM79068	GSM79098	13.60
	GSM79102	GSM79084	16.51
	GSM79102	GSM79098	13.70
	GSM79071	GSM79084	8.11
	GSM79071	GSM79098	6.73
	GSM79103	GSM79100	8.83
	GSM79103	GSM79111	8.36
	GSM79113	GSM79100	4.38
	GSM79113	GSM79111	4.15
PSMA6	GSM79113	GSM79111	2.00
	GSM79074	GSM79112	2.18
	GSM79069	GSM79105	2.40
	GSM79069	GSM79082	2.54
	GSM79069	GSM79084	2.26
	GSM79070	GSM79105	2.62
	GSM79070	GSM79082	2.67
	GSM79070	GSM79084	2.37
	GSM79071	GSM79080	2.06
	GSM79102	GSM79080	2.05

Note: Samples were extracted from GSE3493, “Treatment” and “Control” represent responders and non-responders to preoperative radiotherapy, respectively, Fold-change = Treatment/Control.

Supplementary Table S10: Summary of miRNA expression datasets used in this study

Information	miRNA dataset
GEO Accession	GSE29298
Patients	38
Males	25
Females	13
Good responders	9
Less responding patients	29
Age	64.4 (42.7–79.3)
PMID	22172905
Tissue	fresh biopsies

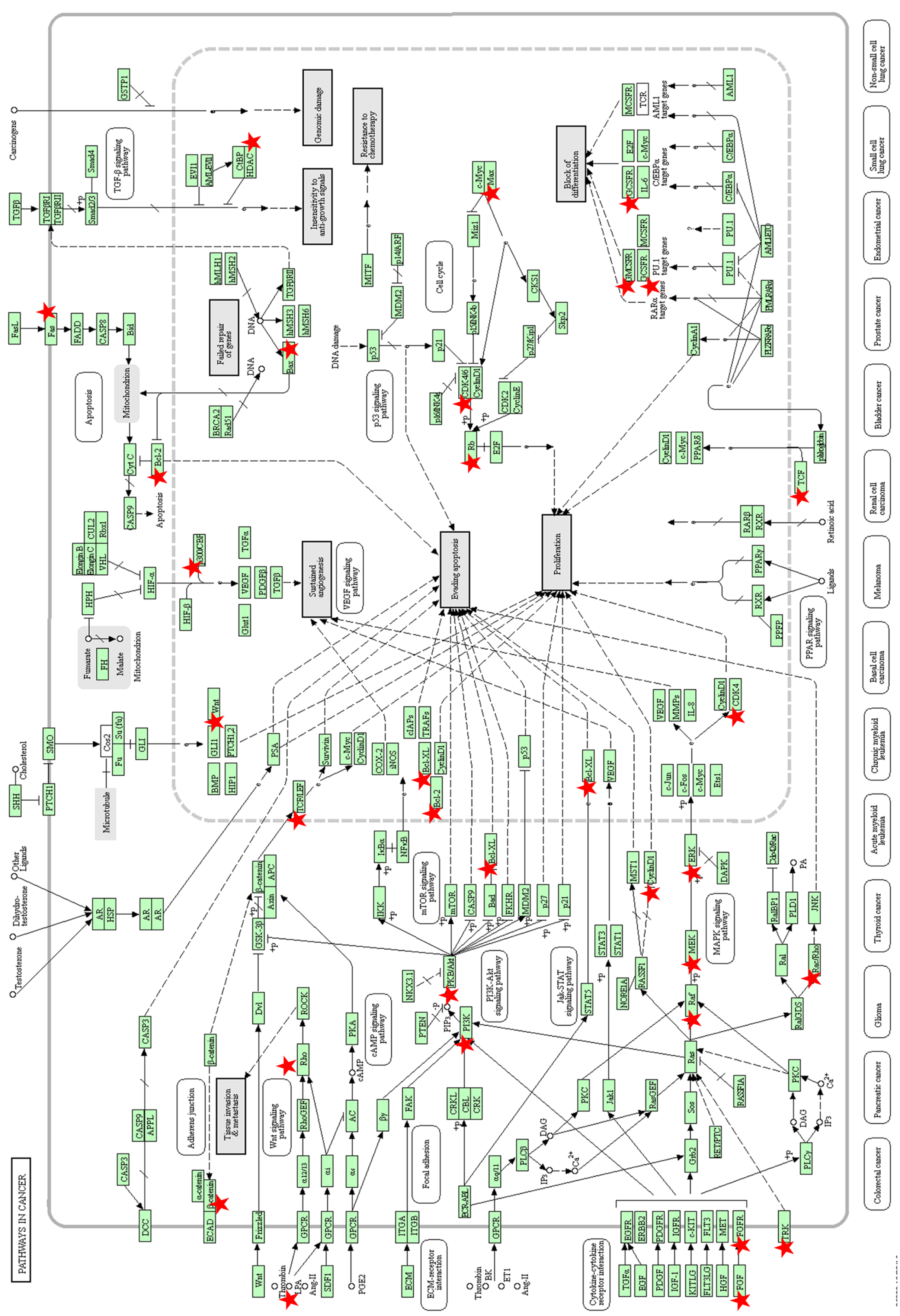

Supplementary Figure S1: Pathways in cancer (from KEGG). This pathway was significantly enriched in the KEGG analysis. Objects with pentagrams were the acting locus by mapped genes.

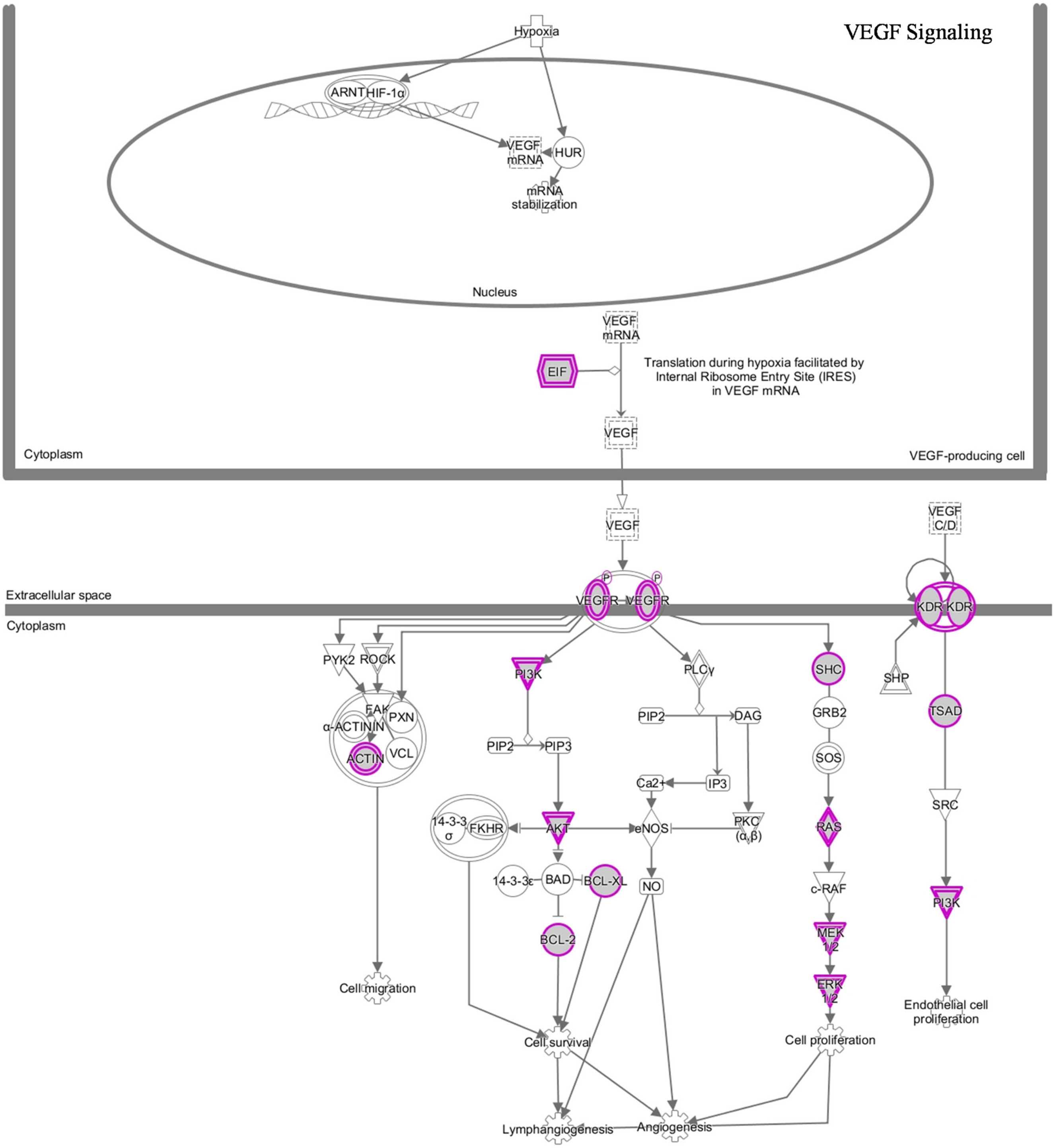

Supplementary Figure S2: VEGF signaling pathway (from IPA). This pathway was significantly enriched in the IPA analysis. Objects with purple circles are the acting locus by mapped genes.

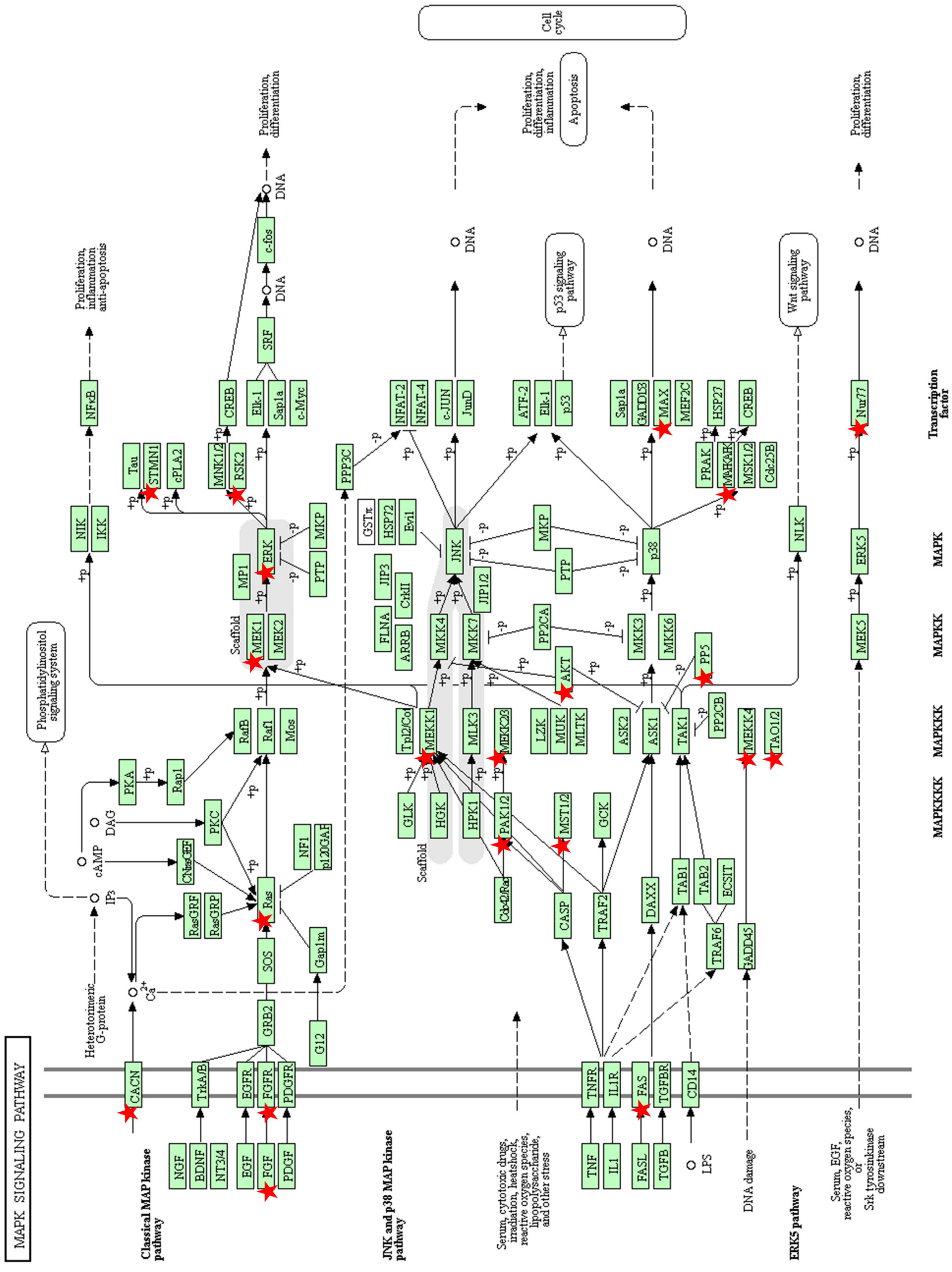

Supplementary Figure S3: MAPK signaling pathway (from KEGG). This pathway was significantly enriched in the KEGG analysis. Objects with pentagrams are the acting locus by mapped genes.
