# Supplementary material for: Identification of biomarker microRNAs for predicting the response of colorectal cancer to neoadjuvant chemoradiotherapy based on microRNA regulatory network
Source: Oncotarget. 2016 Nov 26;8(2):2233–48. doi: 10.18632/oncotarget.13659 (PMC5356795; doi:10.18632/oncotarget.13659)
Supplement: Supplementary file 2 [file oncotarget-08-2233-s002.doc]

**Supplementary Table S3: The miRNA-mRNA pairs in CRC specific miRNA-mRNA network**

| **miRNA ID** | **Gene symbol** |
| --- | --- |
| miR-630 | KIAA1324 |
| miR-630 | MEF2D |
| miR-630 | EP300 |
| miR-630 | BHLHB2 |
| miR-630 | VIL2 |
| miR-630 | SOCS2 |
| miR-630 | ALDOB |
| miR-630 | TEX264 |
| miR-630 | KCNMA1 |
| miR-630 | PTCD2 |
| miR-630 | TJP1 |
| miR-630 | HSPB8 |
| miR-630 | FKBP3 |
| miR-630 | GSTT2 |
| miR-630 | KCNK1 |
| miR-630 | NPAS2 |
| miR-630 | RBMX |
| miR-630 | RBM4 |
| miR-630 | MSMB |
| miR-630 | VPS35 |
| miR-630 | HDAC6 |
| miR-630 | SULT2A1 |
| miR-630 | ZMYM2 |
| miR-630 | KALRN |
| miR-630 | ATP6V0C |
| miR-630 | YIPF6 |
| miR-630 | PIK3R1 |
| miR-630 | SMEK1 |
| miR-630 | MAP3K2 |
| miR-630 | TAC3 |
| miR-630 | FKBP1B |
| miR-630 | PRDM13 |
| miR-630 | TFAP2B |
| miR-630 | PSMA1 |
| miR-630 | SPRY1 |
| miR-630 | SERF2 |
| miR-630 | TAGLN3 |
| miR-630 | ATP1A1 |
| miR-630 | BCOR |
| miR-630 | BCL2 |

*(Continued )*

| **miRNA ID** | **Gene symbol** |
| --- | --- |
| miR-630 | BBOX1 |
| miR-630 | MBNL2 |
| miR-630 | CENTG2 |
| miR-630 | SNTG2 |
| miR-630 | SNTB2 |
| miR-630 | KRT24 |
| miR-630 | KRT83 |
| miR-630 | UTP6 |
| miR-630 | SEMA3D |
| miR-630 | SEMA7A |
| miR-630 | TMEM156 |
| miR-630 | ARMC8 |
| miR-630 | ARL8B |
| miR-630 | PPP2R5B |
| miR-630 | AKAP11 |
| miR-630 | TFG |
| miR-630 | KIAA0146 |
| miR-630 | EZH2 |
| miR-630 | PPP1R3C |
| miR-630 | TBP |
| miR-630 | CASQ1 |
| miR-630 | CSAD |
| miR-630 | CRISP3 |
| miR-630 | PCDH11X |
| miR-630 | YTHDF1 |
| miR-630 | ASPA |
| miR-630 | WTAP |
| miR-630 | NMU |
| miR-630 | LRP6 |
| miR-630 | ARF4 |
| miR-630 | GNL3L |
| miR-630 | CLPS |
| miR-630 | HIST1H2AE |
| miR-630 | TIPRL |
| miR-630 | VTCN1 |
| miR-630 | CLK2 |
| miR-630 | PRG4 |
| miR-630 | CACNA1G |
| miR-630 | LAPTM4A |
| miR-630 | SEC61G |

*(Continued )*

| **miRNA ID** | **Gene symbol** |
| --- | --- |
| miR-630 | CDC27 |
| miR-630 | SNAI2 |
| miR-630 | GALNT1 |
| miR-630 | SENP3 |
| miR-630 | LMNB1 |
| miR-630 | COMMD10 |
| miR-630 | PUM1 |
| miR-630 | VIM |
| miR-630 | KIF2A |
| miR-630 | USP34 |
| miR-630 | ANKHD1 |
| miR-630 | CA2 |
| miR-630 | SPINK5 |
| miR-630 | CBX5 |
| miR-630 | CAST |
| miR-630 | YAP1 |
| miR-630 | HMGCR |
| miR-630 | BCL2L2 |
| miR-630 | DNAJC13 |
| miR-630 | STAG2 |
| miR-630 | TOMM7 |
| miR-630 | FAM12B |
| miR-630 | PLK1 |
| miR-630 | NEO1 |
| miR-630 | FCER1A |
| miR-630 | TDRKH |
| miR-630 | PAWR |
| miR-630 | ZNF187 |
| miR-630 | KDR |
| miR-630 | PAEP |
| miR-630 | ZNF410 |
| miR-630 | ZNF384 |
| miR-630 | IL10 |
| miR-630 | GIPC1 |
| miR-630 | FAM98A |
| miR-630 | ZNF536 |
| miR-630 | PDCL |
| miR-630 | DCP1A |
| miR-630 | ERICH1 |
| miR-630 | CEP192 |

*(Continued )*

| **miRNA ID** | **Gene symbol** |
| --- | --- |
| miR-630 | ZNF711 |
| miR-202-3p | ZMYM2 |
| miR-202-3p | SMAP1 |
| miR-202-3p | SSU72 |
| miR-202-3p | TRIM71 |
| miR-202-3p | NGLY1 |
| miR-202-3p | PVRL3 |
| miR-202-3p | SNX5 |
| miR-202-3p | ZNF193 |
| miR-202-3p | ISG20L2 |
| miR-202-3p | HPCAL1 |
| miR-202-3p | CEP55 |
| miR-202-3p | HSD17B11 |
| miR-202-3p | C1orf43 |
| miR-202-3p | SRGN |
| miR-202-3p | FKRP |
| miR-202-3p | LARP7 |
| miR-202-3p | RPL23A |
| miR-202-3p | TCOF1 |
| miR-202-3p | SLMAP |
| miR-202-3p | H2AFZ |
| miR-202-3p | MTHFD2 |
| miR-202-3p | GABBR1 |
| miR-202-3p | TAF9 |
| miR-202-3p | MTMR4 |
| miR-202-3p | SULF2 |
| miR-202-3p | KLF12 |
| miR-202-3p | SMNDC1 |
| miR-202-3p | DDX50 |
| miR-202-3p | HIGD2A |
| miR-202-3p | MON2 |
| miR-202-3p | POLR3D |
| miR-202-3p | DNAJB11 |
| miR-202-3p | ATXN10 |
| miR-202-3p | RBM9 |
| miR-202-3p | RBM4 |
| miR-202-3p | CHPT1 |
| miR-202-3p | PBX3 |
| miR-202-3p | EEF1A1 |
| miR-202-3p | PANX2 |

*(Continued )*

| **miRNA ID** | **Gene symbol** |
| --- | --- |
| miR-202-3p | ZSWIM4 |
| miR-202-3p | TRIM5 |
| miR-202-3p | FUS |
| miR-202-3p | MYCN |
| miR-202-3p | IRF8 |
| miR-202-3p | C21orf63 |
| miR-202-3p | DBT |
| miR-202-3p | ZBTB10 |
| miR-202-3p | RNPS1 |
| miR-202-3p | PCNA |
| miR-202-3p | C18orf21 |
| miR-202-3p | SLC31A2 |
| miR-202-3p | EDC4 |
| miR-202-3p | SENP2 |
| miR-202-3p | NR2F2 |
| miR-202-3p | TMEM138 |
| miR-202-3p | SF3A3 |
| miR-202-3p | GTPBP2 |
| miR-202-3p | CKAP5 |
| miR-202-3p | HNRPC |
| miR-202-3p | POLQ |
| miR-202-3p | VGLL3 |
| miR-202-3p | BBS10 |
| miR-202-3p | LRRC41 |
| miR-202-3p | IVNS1ABP |
| miR-202-3p | TEX261 |
| miR-202-3p | PYGO2 |
| miR-202-3p | ARMCX5 |
| miR-202-3p | CANT1 |
| miR-202-3p | KLHDC3 |
| miR-202-3p | SMUG1 |
| miR-202-3p | HIPK2 |
| miR-202-3p | SPCS1 |
| miR-202-3p | MAP3K1 |
| miR-202-3p | HTATIP2 |
| miR-202-3p | DNAJC1 |
| miR-202-3p | KLHL15 |
| miR-202-3p | ASXL1 |
| miR-202-3p | RAB2B |
| miR-202-3p | LSM6 |

*(Continued )*

| **miRNA ID** | **Gene symbol** |
| --- | --- |
| miR-202-3p | RNF13 |
| miR-202-3p | C3orf63 |
| miR-202-3p | SLC39A8 |
| miR-575 | SACS |
| miR-575 | MS4A4A |
| miR-575 | RAB6IP1 |
| miR-575 | EHMT2 |
| miR-575 | NR4A1 |
| miR-575 | PLAGL1 |
| miR-575 | PRKAG2 |
| miR-575 | RPS6KA1 |
| miR-575 | RPS6KA3 |
| miR-575 | PIK3CD |
| miR-575 | RAB38 |
| miR-575 | CACNA2D1 |
| miR-575 | FKBP14 |
| miR-575 | GPR63 |
| miR-575 | TIPRL |
| miR-575 | MAP2K1 |
| miR-575 | WDR57 |
| miR-575 | NRP1 |
| miR-575 | SLC14A1 |
| miR-575 | CHD3 |
| miR-575 | RLF |
| miR-575 | KIAA1794 |
| miR-575 | CHRD |
| miR-575 | ENPP2 |
| miR-575 | FGFR2 |
| miR-575 | TMC7 |
| miR-575 | TMEM4 |
| miR-575 | RGS3 |
| miR-575 | PPP1R15A |
| miR-575 | UBAP2 |
| miR-575 | FAS |
| miR-575 | CCNT2 |
| miR-575 | DCHS1 |
| miR-575 | TPT1 |
| miR-575 | HIF3A |
| miR-575 | ANKRD46 |
| miR-575 | ANKRD49 |

*(Continued )*

| **miRNA ID** | **Gene symbol** |
| --- | --- |
| miR-575 | SPINK5 |
| miR-575 | NDUFB1 |
| miR-575 | RBM14 |
| miR-575 | HMGA2 |
| miR-575 | MYO10 |
| miR-575 | TACSTD1 |
| miR-575 | STOML1 |
| miR-575 | MYO5A |
| miR-575 | USP19 |
| miR-575 | VASP |
| miR-575 | MID2 |
| miR-575 | IFNGR2 |
| miR-575 | DPP8 |
| miR-575 | KCTD13 |
| miR-575 | TBC1D17 |
| miR-575 | MRPS7 |
| miR-575 | COX6B1 |
| miR-575 | GALNT1 |
| miR-575 | POLD4 |
| miR-575 | ARPC4 |
| miR-575 | LMBRD1 |
| miR-575 | COPS2 |
| miR-575 | SNRPB |
| miR-575 | FGF1 |
| miR-575 | ARIH2 |
| miR-575 | TUBG1 |
| miR-575 | UCHL5 |
| miR-575 | ACTR3B |
| miR-765 | DTX2 |
| miR-765 | PSMA6 |
| miR-765 | IGSF1 |
| miR-765 | PFKFB3 |
| miR-765 | MAP3K4 |
| miR-765 | SLC25A37 |
| miR-765 | TACC2 |
| miR-765 | PDE1C |
| miR-765 | ST3GAL2 |
| miR-765 | HNF4A |
| miR-765 | GNL3L |
| miR-765 | SV2A |

*(Continued )*

| **miRNA ID** | **Gene symbol** |
| --- | --- |
| miR-765 | BFSP2 |
| miR-765 | BCL6 |
| miR-765 | STK4 |
| miR-765 | BAG1 |
| miR-765 | TAOK3 |
| miR-765 | BAX |
| miR-765 | NRBP1 |
| miR-765 | SCAMP3 |
| miR-765 | CFP |
| miR-765 | IQCB1 |
| miR-765 | FAM117A |
| miR-765 | KLK10 |
| miR-765 | IGFBP2 |
| miR-765 | TAGLN |
| miR-765 | DNAJC7 |
| miR-765 | LIN7B |
| miR-765 | TUBG1 |
| miR-765 | MGA |
| miR-765 | SGCE |
| miR-765 | FKBP1A |
| miR-765 | LBP |
| miR-765 | APOB |
| miR-765 | FUS |
| miR-765 | PIB5PA |
| miR-765 | COLEC12 |
| miR-765 | LMNA |
| miR-765 | LMO1 |
| miR-765 | EZH2 |
| miR-765 | CENPT |
| miR-765 | NECAP2 |
| miR-765 | CSH2 |
| miR-765 | JARID1C |
| miR-765 | MYO1A |
| miR-765 | MYO1C |
| miR-765 | USP19 |
| miR-765 | GAPVD1 |
| miR-765 | PUM1 |
| miR-765 | EIF4G1 |
| miR-765 | NRXN2 |
| miR-765 | NYX |

*(Continued )*

| **miRNA ID** | **Gene symbol** |
| --- | --- |
| miR-765 | BEXL1 |
| miR-765 | EIF2C1 |
| miR-765 | EPHB2 |
| miR-765 | PREB |
| miR-765 | SPINK5 |
| miR-765 | NTRK3 |
| miR-765 | ADIPOR1 |
| miR-765 | PTGDS |
| miR-765 | CYB5A |
| miR-765 | NEU3 |
| miR-765 | SPTLC3 |
| miR-765 | GPC3 |
| miR-765 | NDUFA7 |
| miR-765 | SMARCA4 |
| miR-765 | ACTA2 |
| miR-765 | TRAF3IP2 |
| miR-765 | PVRL1 |
| miR-765 | SH2D2A |
| miR-765 | WHSC1 |
| miR-765 | PCMT1 |
| miR-765 | CACNA1G |
| miR-765 | XPNPEP1 |
| miR-765 | TPM3 |
| miR-765 | AGPAT7 |
| miR-765 | HTRA1 |
| miR-765 | RGS7 |
| miR-765 | SEMA6A |
| miR-765 | PRPF3 |
| miR-765 | OSBPL7 |
| miR-765 | TLE4 |
| miR-765 | HOXC6 |
| miR-765 | HOXB8 |
| miR-765 | KIRREL |
| miR-765 | HIF3A |
| miR-765 | PRR13 |
| miR-765 | RBM4 |
| miR-765 | RALY |
| miR-765 | BST1 |
| miR-765 | TIAL1 |
| miR-765 | CAPN3 |

*(Continued )*

| **miRNA ID** | **Gene symbol** |
| --- | --- |
| miR-765 | CCDC51 |
| miR-765 | PPP4C |
| miR-765 | PPP5C |
| miR-765 | IDH1 |
| miR-765 | COX6B1 |
| miR-765 | TMEM160 |
| miR-513a-5p | LITAF |
| miR-513a-5p | HOXD11 |
| miR-513a-5p | MRPL14 |
| miR-513a-5p | CLK2 |
| miR-513a-5p | KIAA0802 |
| miR-513a-5p | FYN |
| miR-513a-5p | FAM107B |
| miR-513a-5p | WTAP |
| miR-513a-5p | B7-H |
| miR-513a-5p | MBD6 |
| miR-513a-5p | MBIP |
| miR-513a-5p | CD274 |
| miR-513a-5p | TXNRD1 |
| miR-513a-5p | APCDD1 |
| miR-513a-5p | MIA3 |
| miR-513a-5p | EWSR1 |
| miR-513a-5p | HELLS |
| miR-513a-5p | MND1 |
| miR-513a-5p | SLC25A5 |
| miR-513a-5p | PPP1CA |
| miR-513a-5p | SERPINH1 |
| miR-513a-5p | MAPKAPK3 |
| miR-513a-5p | STMN1 |
| miR-198 | RAP1GAP |
| miR-198 | NUMA1 |
| miR-198 | COL5A1 |
| miR-198 | EPS8L3 |
| miR-198 | BCL2L1 |
| miR-198 | FAT |
| miR-198 | COL6A3 |
| miR-198 | BUB3 |
| miR-198 | FUS |
| miR-198 | TARBP2 |
| miR-198 | MARCH2 |

*(Continued )*

| **miRNA ID** | **Gene symbol** |
| --- | --- |
| miR-198 | BNC1 |
| miR-198 | SLC25A28 |
| miR-198 | DCX |
| miR-198 | EPB41L1 |
| miR-198 | RGS10 |
| miR-198 | H3F3A |
| miR-198 | CLU |
| miR-198 | TCTA |
| miR-198 | PIK3C2G |
| miR-198 | TIMP1 |
| miR-198 | RB1 |
| miR-198 | CNOT3 |
| miR-198 | CD160 |
| miR-198 | CCNT1 |
| miR-198 | DPF1 |
| miR-198 | EPHA2 |
| miR-198 | PAPPA |
| miR-198 | ARHGAP19 |
| miR-198 | MLL2 |
| miR-198 | FBXL5 |
| miR-198 | MYB |
| miR-198 | SEMA7A |
| miR-198 | TITF1 |
| miR-198 | DENND2D |
| miR-198 | HOOK2 |
| miR-198 | LBP |
| miR-198 | CDC14A |
| miR-198 | CENPE |
| miR-198 | VAT1 |
| miR-198 | SLC8A1 |
| miR-198 | TXNDC9 |
| miR-198 | PIGC |
| miR-198 | NR2F6 |
| miR-198 | CTNNA3 |
| miR-198 | CD84 |
| miR-198 | FIP1L1 |
| miR-198 | CDK4 |
| miR-198 | CDK6 |
| miR-198 | FAM62A |
| miR-198 | SPRY2 |

*(Continued )*

| **miRNA ID** | **Gene symbol** |
| --- | --- |
| miR-198 | PSMD7 |
| miR-198 | PSME3 |
| miR-198 | PLAU |
| miR-198 | RRAS |
| miR-198 | MBNL2 |
| miR-198 | SLC35A2 |
| miR-198 | NFYA |
| miR-198 | FAM46A |
| miR-198 | SLC16A1 |
| miR-198 | GNB2 |
| miR-198 | GOLGB1 |
| miR-198 | CALU |
| miR-198 | NDRG3 |
| miR-198 | PHKB |
| miR-198 | GNL1 |
| miR-198 | FBLN2 |
| miR-198 | PHF8 |
| miR-198 | RNF4 |
| miR-198 | PAK2 |
| miR-198 | LIN7B |
| miR-198 | TPST2 |
| miR-198 | ANG |
| miR-198 | TMPO |
| miR-198 | CSF2RA |
| miR-198 | PBX1 |
| miR-198 | SAPS1 |
| miR-198 | CKMT1A |
| miR-198 | CELSR2 |
| miR-198 | SNX17 |
| miR-198 | RCE1 |
| miR-198 | RFXANK |
| miR-198 | ATXN1 |
| miR-198 | KRT76 |
| miR-198 | SEC24C |
| miR-198 | SEC24B |
| miR-198 | IL10 |
| miR-198 | TLE4 |
| miR-198 | TCF7L1 |
| miR-198 | CTSC |
| miR-198 | CTNNBL1 |

*(Continued )*

| **miRNA ID** | **Gene symbol** |
| --- | --- |
| miR-198 | APH1A |
| miR-198 | APEH |
| miR-198 | ZNF281 |
| miR-198 | ZNF289 |
| miR-198 | IL11RA |
| miR-198 | ARMC8 |
| miR-198 | HIVEP2 |
| miR-198 | PCDH12 |
| miR-198 | PLXNA1 |
| miR-198 | VPS35 |
| miR-198 | NCKAP1L |
| miR-198 | IFNGR2 |
| miR-198 | EIF4E2 |
| miR-198 | ARID1A |
| miR-198 | NDUFC1 |
| miR-198 | GAPVD1 |
| miR-198 | ALPL |
| miR-198 | TFCP2 |
| miR-198 | UFC1 |
| miR-198 | GPRC5C |
| miR-198 | ZCCHC4 |
| miR-198 | H2AFX |
| miR-198 | NTRK3 |
| miR-198 | AIM1L |
| miR-198 | CLTA |
| miR-198 | CRISP3 |
| miR-198 | GZMH |
| miR-198 | JAM2 |
| miR-198 | PGAP1 |
| miR-198 | PTMS |
| miR-198 | SMC1A |
| miR-198 | NOL3 |
| miR-198 | SLITRK5 |
| miR-198 | CHD1 |
| miR-198 | CHD4 |
| miR-198 | ACHE |
| miR-198 | ACE2 |
| miR-198 | ATG9A |
| miR-198 | HPRT1 |
| miR-198 | ATG16L1 |

*(Continued )*

| **miRNA ID** | **Gene symbol** |
| --- | --- |
| miR-198 | DHRS1 |
| miR-198 | PPA2 |
| miR-198 | SRGAP2 |
| miR-198 | RBM12 |
| miR-198 | ADH5 |
| miR-198 | RBM4B |
| miR-198 | WNT7A |
| miR-198 | BAI3 |
| miR-198 | CXCR7 |
| miR-198 | MAP4 |
| miR-198 | HABP2 |
| miR-198 | c-Myb |
| miR-198 | USP16 |
| miR-198 | PTPRD |
| miR-198 | BCL9 |
| miR-198 | WAC |
| miR-198 | SH3GLB1 |
| miR-198 | LASS4 |
| miR-198 | TAZ |
| miR-198 | STAU2 |
| miR-198 | DDR2 |
| miR-198 | SCD |
| miR-198 | PPP2CB |
| miR-198 | DFNB31 |
| miR-198 | PCDH1 |
| miR-198 | UBE2M |
| miR-198 | PKD2L2 |
| miR-198 | URM1 |
| miR-198 | ITGB4BP |
| miR-198 | RIMS3 |
| miR-198 | CDK2AP2 |
| miR-198 | PLAUR |
| miR-198 | NXF1 |
| miR-198 | ARAF |
| miR-198 | ARL6IP1 |
| miR-198 | SGSH |
| miR-198 | FABP5 |
| miR-198 | SHC1 |
| miR-198 | ZDHHC6 |
| miR-198 | DCTN3 |

*(Continued )*

| **miRNA ID** | **Gene symbol** |
| --- | --- |
| miR-198 | TBPL1 |
| miR-198 | M6PR |
| miR-198 | CXCL13 |
| miR-483-5p | RUSC2 |
| miR-483-5p | FBN2 |
| miR-483-5p | NRBP1 |
| miR-483-5p | SILV |
| miR-483-5p | ARHGEF9 |
| miR-483-5p | ZNF289 |
| miR-483-5p | PPP2R1A |
| miR-483-5p | SHOX2 |
| miR-483-5p | ALDOC |
| miR-483-5p | TFDP1 |
| miR-483-5p | PABPN1 |
| miR-483-5p | LYPLA2 |
| miR-483-5p | VAMP8 |
| miR-483-5p | FHL1 |
| miR-483-5p | DBN1 |
| miR-483-5p | CDKL3 |
| miR-483-5p | PREP |
| miR-483-5p | B4GALT3 |
| miR-483-5p | RBM35A |
| miR-483-5p | SAMD4A |
| miR-483-5p | JARID1C |
| miR-483-5p | TFAP2B |
| miR-483-5p | CHD1 |
| miR-483-5p | NFX1 |
| miR-483-5p | SMARCA2 |
| miR-483-5p | UBE2D2 |
| miR-483-5p | H2AFV |
| miR-483-5p | CTSK |
| miR-483-5p | SH3GL3 |
| miR-483-5p | PDGFD |
| miR-483-5p | APBB2 |
| miR-483-5p | ZCWPW1 |
| miR-483-5p | CACNB1 |
| miR-483-5p | KLHDC3 |
| miR-483-5p | MRPL52 |
| miR-483-5p | UCK2 |
| miR-483-5p | DNAJC7 |

*(Continued )*

| **miRNA ID** | **Gene symbol** |
| --- | --- |
| miR-483-5p | PIP5K1A |
| miR-483-5p | RFC3 |
| miR-483-5p | MTHFD2L |
| miR-483-5p | MAN1A2 |
| miR-483-5p | GCC2 |
| miR-483-5p | SLC25A15 |
| miR-483-5p | RBM5 |
| miR-483-5p | RGS12 |
| miR-483-5p | RNF10 |
| miR-483-5p | GNLY |
| miR-483-5p | NAB2 |
| miR-483-5p | CD53 |
| miR-483-5p | AP2M1 |
| miR-483-5p | RHOA |
| miR-483-5p | MON1B |
| miR-483-5p | ADRBK1 |
| miR-483-5p | FOXJ2 |
| miR-483-5p | SLC23A2 |
| miR-483-5p | MYO9B |
| miR-483-5p | USP48 |
| miR-483-5p | BCAM |
| miR-483-5p | BAG1 |
| miR-483-5p | MEA1 |
| miR-483-5p | CITED2 |
| miR-483-5p | PDE4DIP |
| miR-483-5p | PRIM2A |
| miR-483-5p | MLL |
| miR-483-5p | MAX |
| miR-483-5p | MAPK3 |
| miR-483-5p | CD320 |
| miR-483-5p | SON |
| miR-483-5p | SET |
| miR-483-5p | UBE2Z |
| miR-483-5p | DNMT1 |
| miR-483-5p | CAMK2G |
| miR-483-5p | DRG1 |
| miR-483-5p | ISGF3G |
| miR-371a-5p | BAG3 |
| miR-371a-5p | ZNF564 |
| miR-371a-5p | ZNF503 |

*(Continued )*

| **miRNA ID** | **Gene symbol** |
| --- | --- |
| miR-371a-5p | TBP |
| miR-371a-5p | ZNF644 |
| miR-371a-5p | HTRA1 |
| miR-371a-5p | SPTBN1 |
| miR-371a-5p | SEC23A |
| miR-371a-5p | SLC35A5 |
| miR-371a-5p | KIAA0907 |
| miR-371a-5p | PSMC6 |
| miR-371a-5p | ENOPH1 |
| miR-371a-5p | BLMH |
| miR-371a-5p | MLL2 |
| miR-371a-5p | C9orf23 |
| miR-371a-5p | WBP11 |
| miR-371a-5p | CAMK2G |
| miR-371a-5p | DNER |
| miR-371a-5p | MAT2B |
| miR-371a-5p | ZNHIT3 |
| miR-371a-5p | GEMIN5 |
| miR-371a-5p | VPS37C |
| miR-371a-5p | HBS1L |
| miR-371a-5p | BTG3 |
| miR-371a-5p | CNOT3 |
| miR-371a-5p | CITED2 |
| miR-371a-5p | ITGB8 |
| miR-371a-5p | BAZ1A |
| miR-371a-5p | DCP1A |
| miR-371a-5p | MYCN |
| miR-371a-5p | TLE4 |
| miR-371a-5p | CCNC |
| miR-371a-5p | ZDHHC13 |
| miR-371a-5p | ZDHHC23 |
| miR-371a-5p | CBX4 |
| miR-371a-5p | TUBB |
| miR-371a-5p | PHF2 |
| miR-371a-5p | TTF1 |
| miR-371a-5p | CALU |
| miR-371a-5p | NME4 |
| miR-371a-5p | NDFIP1 |
| miR-371a-5p | HMGN2 |
| miR-371a-5p | AAMP |

*(Continued )*

| **miRNA ID** | **Gene symbol** |
| --- | --- |
| miR-371a-5p | NGRN |
| miR-371a-5p | CKS2 |
| miR-371a-5p | LEF1 |
| miR-371a-5p | EPN1 |
| miR-371a-5p | GHITM |
| miR-371a-5p | NUF2 |
| miR-371a-5p | GSR |
| miR-371a-5p | GLRX2 |
| miR-371a-5p | LRRC42 |
| miR-371a-5p | COPS4 |
| miR-371a-5p | CSNK1E |
| miR-371a-5p | CR2 |
| miR-371a-5p | YTHDF1 |
| miR-371a-5p | SIPA1L2 |
| miR-371a-5p | TTC21B |
| miR-371a-5p | GOLGA7 |
| miR-371a-5p | BECN1 |
| miR-371a-5p | INTS3 |
| miR-371a-5p | CTNNA1 |
| miR-371a-5p | UNKL |
| miR-671-5p | SELP |
| miR-671-5p | LRP8 |
| miR-671-5p | PIP5K2C |
| miR-671-5p | ESRRA |
| miR-671-5p | PIP5K3 |
| miR-671-5p | ATF3 |
| miR-671-5p | SEPT6 |
| miR-671-5p | HLA-DRB1 |
| miR-671-5p | PRPF31 |
| miR-671-5p | GPR125 |
| miR-671-5p | FGL1 |
| miR-671-5p | SCMH1 |
| miR-671-5p | NOS1 |
| miR-671-5p | SPTBN1 |
| miR-671-5p | SPTBN2 |
| miR-671-5p | AKT2 |
| miR-671-5p | SATB2 |
| miR-671-5p | DNAJB5 |
| miR-671-5p | BTNL8 |
| miR-671-5p | HCN2 |

*(Continued )*

| **miRNA ID** | **Gene symbol** |
| --- | --- |
| miR-671-5p | COX5A |
| miR-671-5p | UBAP2L |
| miR-671-5p | SSBP1 |
| miR-671-5p | RNF38 |
| miR-671-5p | RAB13 |
| miR-671-5p | ADD1 |
| miR-671-5p | TSPYL2 |
| miR-671-5p | DCTN2 |
| miR-671-5p | PSG1 |
| miR-671-5p | EIF2B3 |
| miR-671-5p | CD34 |
| miR-671-5p | PHF15 |
| miR-671-5p | PKN2 |
| miR-671-5p | ELP4 |
| miR-671-5p | MYST1 |
| miR-671-5p | TIPARP |
| miR-671-5p | SF1 |
| miR-671-5p | CLINT1 |
| miR-671-5p | CLEC4E |
| miR-671-5p | YWHAQ |
| miR-671-5p | IL10 |
| miR-671-5p | BSDC1 |
| miR-671-5p | RAP1GDS1 |
| miR-671-5p | LYZL6 |
| miR-671-5p | MYL6 |
| miR-671-5p | MAX |
| miR-671-5p | VSIG4 |
| miR-671-5p | CAPN6 |
| miR-671-5p | MATN4 |
| miR-671-5p | CSF3R |
| miR-671-5p | DVL3 |
| miR-671-5p | ANKRD25 |
| miR-671-5p | KLF6 |
| miR-671-5p | CSNK2A2 |
| miR-671-5p | IGF2 |
| miR-671-5p | KCNQ3 |
| miR-671-5p | COL16A1 |
| miR-671-5p | HSPH1 |
| miR-671-5p | IL17RB |
| miR-671-5p | HDAC1 |

*(Continued )*

| **miRNA ID** | **Gene symbol** |
| --- | --- |
| miR-671-5p | HMGA2 |
| miR-671-5p | SPTB |
| miR-671-5p | HRH3 |
| miR-671-5p | GON4L |
| miR-671-5p | GFRA1 |
| miR-671-5p | RHOBTB2 |
| miR-671-5p | TRIP13 |
| miR-671-5p | PTGDS |
